# Safety and Efficacy of Zero Fluoroscopy Patent Ductus Arteriosus Closure in Comparison to the Standardized Fluoroscopy-Guided Procedure: A Systematic Review and Meta-Analysis

**DOI:** 10.2174/011573403X338573241101092849

**Published:** 2025-01-31

**Authors:** Brian Mendel, Kelvin Kohar, Richie Jonathan Djiu, Defin Allevia Yumnanisha, Ananda Pipphali Vidya, Justin Winarta, Karunia Hafifah Arifin, Muhammad Dzaky Erlangga Mumtaz, Aqilla Katrita Zaira Nugroho, Gusti Ngurah Prana Jagannatha, Sisca Natalia Siagian, Radityo Prakoso

**Affiliations:** 1 Division of Pediatric Cardiology and Congenital Heart Disease, Department of Cardiology and Vascular Medicine, National Cardiovascular Center Harapan Kita, Universitas Indonesia, Jakarta, Indonesia;; 2 Department of Cardiology and Vascular Medicine, Sultan Sulaiman Government Hospital, Serdang Bedagai, North Sumatera, Indonesia;; 3 Faculty of Medicine, Universitas Indonesia, Jakarta, Indonesia;; 4 Faculty of Medicine, Udayana University, Prof. Dr. I.G.N.G. Ngoerah General Hospital, Denpasar, Bali, Indonesia

**Keywords:** Echocardiography, PDA, percutaneous, TEE, TTE, zero-fluoroscopy

## Abstract

**Background:**

Patent Ductus Arteriosus (PDA) is a common condition in premature infants requiring intervention to avoid problems. Despite improvements in lowering radiation exposure and employing better contrast agents, fluoroscopy is still the most widely employed technique, which exposes interventional echocardiographers to radiation risks. Techniques, such as Transthoracic Echocardiography (TTE)-guided procedures or Transesophageal Echocardiography (TEE)-guided procedures, provide radiation-free options. This systematic review and meta-analysis aimed to evaluate the safety and effectiveness of fluoroscopy-guided versus non-fluoroscopy-guided PDA closure techniques with respect to the reduction in procedural risks and improved clinical decision-making when treating hemodynamically severe PDAs in premature newborns. As there is no specific age or cutoff for this procedure, it is crucial to perform it as early as possible to prevent complications, especially if symptoms are already present.

**Methods:**

This systematic review has been registered in PROSPERO with registration number CRD42024516321. Three electronic databases (PubMed, Scopus, and Google Scholar) have been reviewed up to February 2024 to search the literature. The main outcome has been the procedural success rate. The additional outcomes have included procedural-related complications rate. We have performed a proportional meta-analysis using the random-effects model and the DerSimonian-Laird method. The risk of bias in all included studies has been evaluated using the STROBE guideline.

**Results:**

A total of 85 (78 fluoroscopy and 7 zero-fluoroscopy) studies have been included in this study. Percutaneous PDA closure success rate has been significantly higher in zero-fluoroscopy group compared to fluoroscopy guidance [99.4% (95%CI: 98.1-100%) and 94.6% (95%CI: 92.3-97%, test for subgroup differences *p* < 0.01), respectively]. The complication rate has been similar in both groups [4% (95%CI: 0-10%) in zero-fluoroscopy and 8.9% (95%CI: 6.5-11.3%) in fluoroscopy group, test for subgroup differences; *p* = 0.14]. Device embolization has been the most common complication reported in the fluoroscopy group [1.7% patients (95%CI: 1.1-2.3%)]. Meanwhile, the residual leak has been the only complication reported in the zero-fluoroscopy group [15.6% patients (95%CI: 0-37.5%)].

**Conclusion:**

Patent Ductus Arteriosus (PDA) is common in preemies and requires intervention. While fluoroscopy is widely used with lower radiation and better contrast agents, it still carries radiation risks. Thus, this review has evaluated the safety and effectiveness of fluoroscopy versus zero-fluoroscopy-guided PDA closures, aiming to reduce procedural risks and enhance clinical decisions for treating PDA.

Zero fluoroscopy techniques for percutaneous PDA closure have been found to yield comparable success rates and procedural outcomes to fluoroscopy-guided procedures. Considering its reduced side effects, zero-fluoroscopy is safe and can be the preferred method to guide closures. However, future randomized controlled trials are necessary to better understand the exact role of interventional echocardiography in PDA closures.

## 1 INTRODUCTION

Prematurely born infants often face a common cardiovascular issue known as Patent Ductus Arteriosus (PDA). If left uncorrected, this defect may persist until adulthood. Over the last decade, there has been a significant expansion in percutaneous procedures aimed at treating PDA. Typically, PDA closure is guided by fluoroscopy and Transesophageal Echocardiography (TEE), necessitating the involvement of an interventional echocardiographer in the procedure team. Fluoroscopy-guided transcatheter PDA closure itself has been the preferred method for the past forty years because it allows visualization of the wire and device placement [1,2]. Effective management, timing, and methods of treating PDA have been the subject of plenty of debates, especially considering the array of available treatment options. This poses a challenge in making informed decisions based on evidence after diagnosing hemodynamically significant PDA [3].

Due to the intermittent need for manipulation of the TEE probe during the procedure, the interventional echocardiographer is required to be in close proximity to the patient, which exposes them to scatter radiation, the primary source of radiation during fluoroscopically guided procedures [4]. Prolonged exposure to scatter radiation in the cardiac catheterization laboratory has been linked to various adverse health effects, such as premature cataract development, early carotid atherosclerosis, and potentially, malignant tumors on the left side of the brain [1]. In addition, fluoroscopy increases the overall risk of developing various cancers, leukemia, lymphoma, and solid cancer [5]. With the increasing number of procedures being performed, there is a need to quantify the procedural risk to facilitate informed efforts to mitigate it. While modern techniques allow for reduced fluoroscopic exposure and the use of nonionic contrast agents, the possibility of injury cannot be disregarded, particularly for physicians and patients with renal issues or allergies. The pioneering work by Pan et al., who reported the first transcatheter PDA closure under Transthoracic Echocardiography (TTE) guidance without fluoroscopy, offers promise in addressing the radiation concern [6].

The quandary lies in whether to remove the PDA at all, and if so, what modality should be employed, *i.e*., fluoroscopy-guided or non-fluoroscopy-guided. Consequently, a comprehensive systematic review and meta-analysis was undertaken to consolidate evidence from clinical trials comparing these modalities for treating hemodynamically significant PDAs. The primary objective of this study was to assess the success rate of percutaneous PDA closure by comparing fluoroscopy-guided and zero-fluoroscopy-guided techniques. The secondary objectives were to compare the two groups' overall complications and specific complications, such as device embolization, arrhythmia, and residual leak.

## 2 MATERIALS AND METHODS

### 2.1 Study Design

This systematic review and meta-analysis was conducted following the Preferred Reporting Items for Systematic Review and Meta-Analysis (PRISMA) guidelines [7]. The protocol of this study has been registered in the International Prospective Register of Systematic Reviews (PROSPERO) with registration number CRD42024516321.

### 2.2 Search Strategy

All authors comprehensively reviewed three electronic databases, including PubMed, Scopus, and Google Scholar up to February 15, 2024. Manually listed references were also checked for relevant papers. The following keywords were used: “Patent ductus arteriosus” OR “PDA” OR “Patency of the ductus arteriosus” AND “Percutaneous closure” OR “Transcatheter closure”. Fig. (**1**) shows the PRISMA flow diagram.

### 2.3 Eligibility Criteria and Selection Process

The inclusion criteria of this systematic review and meta-analysis were structured in accordance with the PICO framework, including (1) the type of study: observational study and clinical trial; (2) the study population: patients with patent ductus arteriosus with no specific age range; (3) the intervention: percutaneous closure; and (4) outcome: success rate (defined as successful device placement), complications, and mortality. Studies were excluded if they were non-human studies or experimental, had inaccessible full-text, and were in non-English language. Any discrepancies were resolved by other authors’ judgment. All processes were recorded in Google Sheets.

### 2.4 Data Extraction

Five authors performed data extraction independently using a standard tabulation table. All processes were recorded and performed using Google Sheets. The following details were included: study characteristics (author, year, study type, location, study interval, and follow-up period); baseline characteristics (number of patients, age, sex, weight, and PDA characteristics); device characteristics (size and details); and procedure (guiding closure method, success rate, and complications). The other remaining authors checked the data accuracy.

### 2.5 Quantitative Data Analysis

All data have been reported as a proportion to quantify event occurrence in each individual study. The analysis was performed using RStudio 2202.07.2+576. The proportional meta-analysis, reported as overall proportion, was performed using a random-effects model and the DerSimonian-Laird method. Subgroup analysis was also performed if applicable. Statistical heterogeneity was assessed using I^2^ statistics, ranging from 0% to 100%. We have considered I2 <25% as low heterogeneity, 25-50% as moderate heterogeneity, and >50% as high heterogeneity. A contour-enhanced funnel plot was built to assess publication bias, with Egger’s test linear regression employed for objective assessment. The success rate data were taken immediately after the procedure was performed, defined as successful device placement. In addition, subgroup analysis was performed according to the guiding closure method.

### 2.6 Risk of Bias Assessment

All independent investigators assessed the quality and possible bias of included articles using Strengthening the Reporting of Observational Studies in Epidemiology (STROBE-II) [8]. Potential biases were assessed for information bias, selection bias, and confusion bias. The outcome was divided into green, yellow, and red colors indicating low, some concerns, and high risk of bias, respectively.

## 3 RESULTS

### 3.1 Study Selection

#### 3.1.1 Study Characteristics

A total of 86 studies across the world from 1996 to 2023 were included in this systematic review and meta-analysis. Most included studies were cohort studies (83 studies) and the remaining three were clinical trials. The baseline characteristics of each study are depicted in the supplementary table. In this meta-analysis, we have included all PDA types according to Krichenko's angiographic classification, consisting of types A, B, C, D, and E. PDA type A is the most commonly reported type throughout studies. Percutaneous PDA closure was performed in patients ranging in age from 3 months to 23 years.

A number of 10,275 patients underwent percutaneous PDA closure, with 9,951 patients using fluoroscopy guidance and 324 using zero-fluoroscopy guidance. The devices used varied across the study, such as Amplatzer Duct Occluder (the most commonly used), detachable cook PDA coils, Rashkind PDA occluder, Gianturco coil, Nit-Occlud coil, and others. The mean follow-up period ranged up to 41 months.

#### 3.1.2 Synthesis of Results

78 studies (9,951 patients) performed percutaneous PDA closure guided by fluoroscopy and 7 studies (324 patients) conducted PDA closure guided by zero-fluoroscopy. A study by Chamie *et al*. (2013) only reported the procedure as successful and safe, without the real number to be analyzed. Overall, PDA closure was successfully performed in 95.1% of the patients (95%CI: 92.9-97.2%), as depicted in Fig. (**2**). The procedure success rate was significantly higher in zero-fluoroscopy compared to the fluoroscopy approach [99.4% (95%CI: 98.1-100%) and 94.6% (95%CI: 92.3-97%, test for subgroup differences; *p* < 0.0^1^]. High heterogeneity was found in the fluoroscopy group (I^2^ = 89%), while the zero-fluoroscopy group revealed homogeneity (I^2^ = 0%). The publication bias of this meta-analysis was assessed using a funnel plot and objectively using Egger’s test. The funnel plot showed an asymmetrical representation of studies and Egger’s test revealed *p* < 0.01, indicating publication bias.

**Fig. (3):**
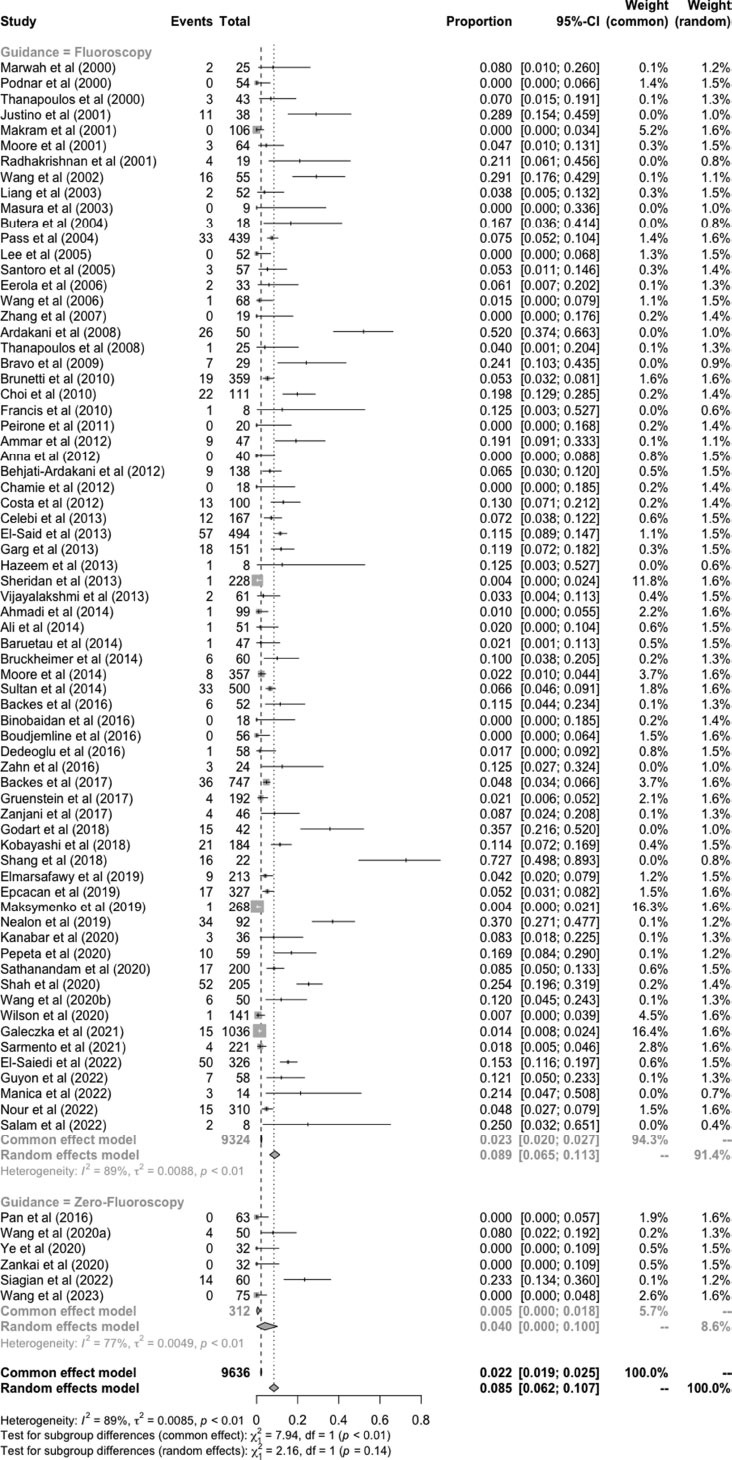
Overall complications of transcatheter PDA closure.

**Fig. (4):**
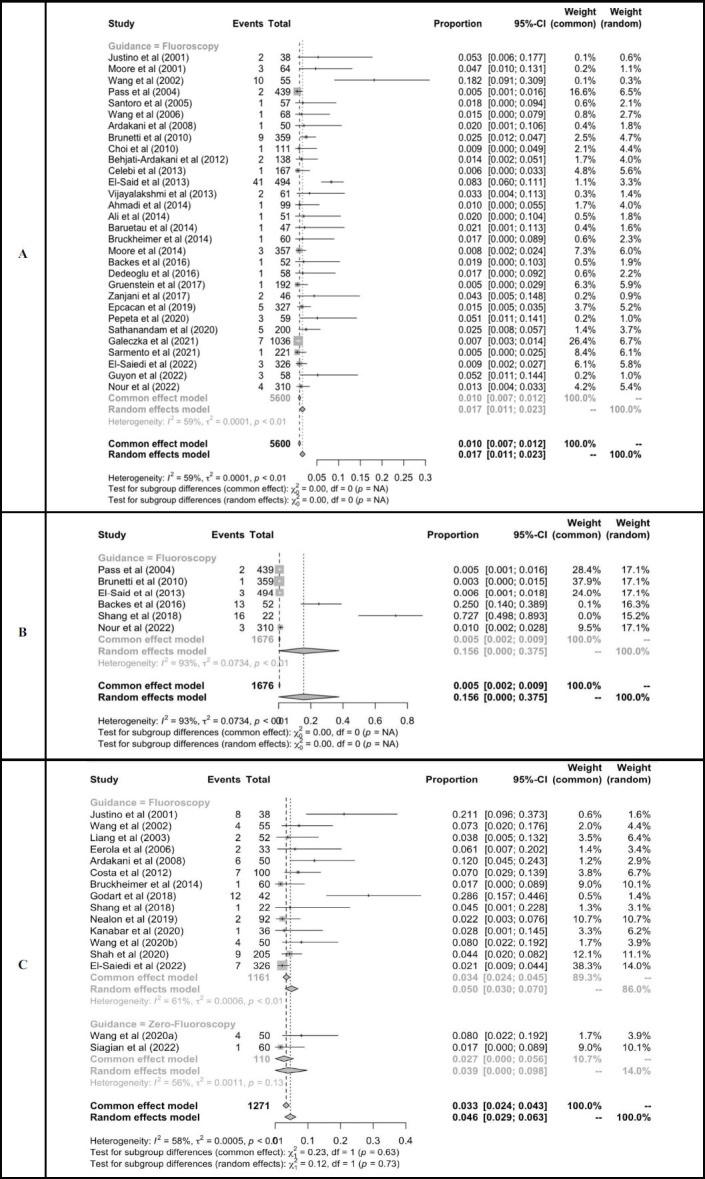
Proportional meta-analysis of complications: device embolization (**A**), arrhythmia (**B**), and residual leak (**C**).

Overall complications related to the procedure were reported in most studies (Fig. **3**). PDA closure guided without fluoroscopy was non-significantly associated with lower complications compared to fluoroscopy groups [4% (95%CI: 0-10%) and 8.9% (95%CI: 6.5-11.3%), test for subgroup differences; *p* = 0.^14^]. Device embolization was the most frequent complication reported in the fluoroscopy group, presenting in 1.7% of the patients (95%CI: 1.1-2.3%). Arrhythmia was observed only in 15.6% of the patients (95%CI: 0-37.5%) in the fluoroscopy group. In the zero-fluoroscopy group, residual leak occurred in 3.9% of the patients (95%CI: 0-9.8%). This number was non-significantly lower compared to the fluoroscopy group, which accounted for 5% of the patients (95%CI: 3-7%) (Fig. **4**).

## 4 DISCUSSION

Persistent ductal shunting can result in pulmonary overcirculation [9], elevating the risk of bronchopulmonary dysplasia. Conversely, this shunting can cause systemic hypoperfusion, which increases the risk of necrotizing enterocolitis, intraventricular hemorrhage, renal failure [9], and death [10]. To address these issues, numerous efforts have been made to remove the Persistent Ductus Arteriosus (PDA) and prevent further complications. Presently, the primary methods for treating PDA in infants, children, and adults include open surgery, percutaneous closure [9, 11, 12] under fluoroscopy guidance, thoracoscopic PDA closure, and transthoracic PDA closure *via* TTE guidance. In a multicenter cohort study by Qian *et al.* (2024) involving 2494 extremely preterm infants diagnosed with PDA, treatment was significantly associated with a reduced risk of death. This positive effect of PDA treatment on mortality was primarily observed in extremely preterm infants who required invasive ventilation [13].

Recently, there has been an increase in reports of PDA closure performed through percutaneous fluoroscopy guidance. However, fluoroscopy and contrast agents have less favorable effects [14], especially for vulnerable populations, such as infants, pregnant women, and patients with kidney disease [1, 15]. Consequently, significant efforts have been made to develop safer techniques [16] for these vulnerable groups. One such technique is zero fluoroscopy, which does not use fluoroscopic radiation or any contrast agents. This method relies solely on transthoracic and/or transesophageal echocardiography guidance, making it theoretically safe for vulnerable populations [17].

Although numerous studies have demonstrated the use of zero fluoroscopy techniques in patients with structural heart disease, such as atrial septal defect, ventricular septal defect, and patent ductus arteriosus closure, most of these studies have been observational and lacked randomized controlled trials [2, 18]. The 2024 SCAI guidelines recommend minimizing radiation exposure to prevent stochastic and deterministic effects on patients. Ironically, the use of zero fluoroscopy for structural heart disease closure is not included in these guidelines [19]. Additionally, interventional echocardiographers, who are exposed to high doses of scattered radiation [20-23] during fluoroscopy procedures, may greatly benefit from zero fluoroscopy techniques. This approach could also be highly advantageous for both patients and operators alike.

The possibility of minimally occluding PDA through the femoral vein under TTE and/or TEE guidance remains a topic of debate [3]. Currently, TTE and/or TEE-guided transcatheter femoral vein occlusion of PDA has shown promising clinical outcomes [12, 18, 24]. In an initial study by Pan *et al.* (2016), 63 consecutive PDA patients underwent transthoracic echocardiography-guided closure, with 62 patients successfully undergoing percutaneous zero fluoroscopy closure. One patient required conversion to minimally invasive transthoracic occlusion due to the failure of the delivery sheath to pass through a tortuous PDA. At a mean follow-up of 13.5 ± 4.8 months, there were no instances of occluder migration, hemolysis, pericardial effusion, or pulmonary branch or aortic stenosis [6]. In another study by Wang *et al.* (2023), 75 children underwent PDA closure through the right femoral vein under TEE guidance. The longest procedural time at the initial stage was 2 hours, and the shortest was 20 minutes. They noted that procedures exceeding 2 hours might need to transition to fluoroscopy-guided or open-heart surgery to mitigate the risks associated with prolonged catheterization. This method has been reported to effectively occlude PDA through the femoral vein without using contrast agents and X-rays, demonstrating both safety and efficacy with no severe complications reported [25].

However, this technique has limitations. First, TEE guidance requires highly technical skills [26]. Unlike fluoroscopy, which easily determines the catheter's location through projection, echocardiography detects it by facets and cannot accurately show the catheter's position [3]. Second, TEE-guided percutaneous interventions present significant technical challenges and a steep learning curve [27]. Without fluoroscopy guidance, the surgeon's spatial perception and timely communication with the sonographer are particularly crucial [26].

In another study by Wang et al., the success rate of percutaneous PDA occlusion using TTE guidance alone was 98% in 50 subjects, comparable to that of fluoroscopy guidance. One patient required conversion to a TEE-guided procedure due to a poor acoustic window, and the occluder was successfully implanted. The findings have further supported the use of TTE guidance. First, the data have indicated PDA occlusion under TTE guidance to significantly reduce total costs compared to fluoroscopy guidance. Since the hospital stay has not differed significantly between the two groups, the cost savings have been primarily attributed to reduced expenses for tools, equipment depreciation, and contrast agents. Second, the procedural time for occlusion under TTE guidance has been shorter than that under fluoroscopy guidance. In their center, zero fluoroscopy guidance has been successfully and routinely implemented by experienced operators, contributing significantly to the favorable procedural times for the TTE guidance group [25].

In this study, we have also found high heterogeneity [I^2^] and an asymmetrical funnel plot, which have implied a high risk of publication bias. However, a study by Barker *et al.* stated that high heterogeneity is a natural and common finding in the proportional meta-analysis, although only a slight variance of real data is observed. Therefore, the results should not be immediately considered as inconsistent. Barker *et al.* also recommended performing qualitative assessment through published data to assess publication bias. In this study, the authors considered publication bias to be minimal because all published data regarding the success rate of transcutaneous PDA closure consistently showed the same number (>85%) [28].

## 5 LIMITATIONS

Given that percutaneous zero fluoroscopy PDA closure is a relatively recent development, the meta-analysis has faced a sample size imbalance, which may have limited the ability to detect significant differences between the two groups. Additionally, all included studies have been observational. Thus, this design may have inherently carried a higher risk of confounding factors, where variables outside of the study’s focus could have influenced the outcomes. Additionally, observational studies are susceptible to selection bias, where the process of choosing participants may lead to a non-representative sample, further impacting the validity of the findings. Moreover, individual institutions may likely employ varied strategies for intervention and reporting, further complicating comparisons.

## CONCLUSION

Percutaneous zero fluoroscopy PDA closure techniques have demonstrated success rates and procedural outcomes comparable to those of fluoroscopy-guided procedures. Future randomized controlled trials are necessary to further elucidate the role of interventional echocardiography in PDA closure, as recommended by the American Society of Echocardiography (ASE). Additionally, studies involving a larger population and comparative analyses between zero-fluoroscopy and traditional fluoroscopy techniques are essential. Such research could enhance the validity and practicality of this method for future PDA closures, ultimately reducing radiation exposure.

## Figures and Tables

**Fig. (1):**
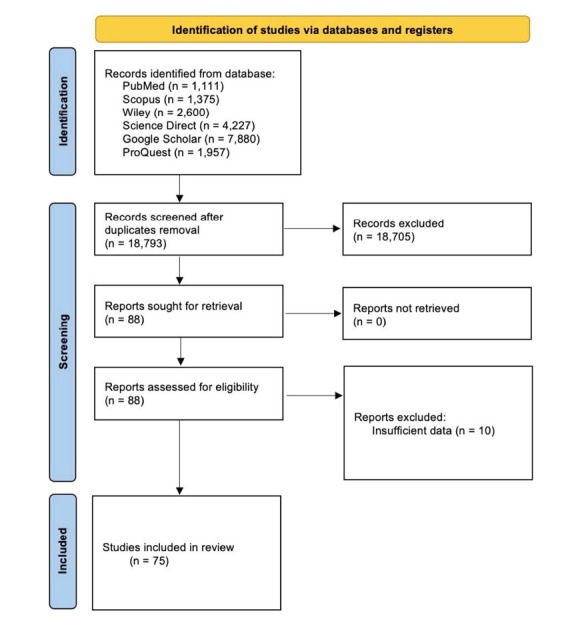
PRISMA flowchart.

**Fig. (2):**
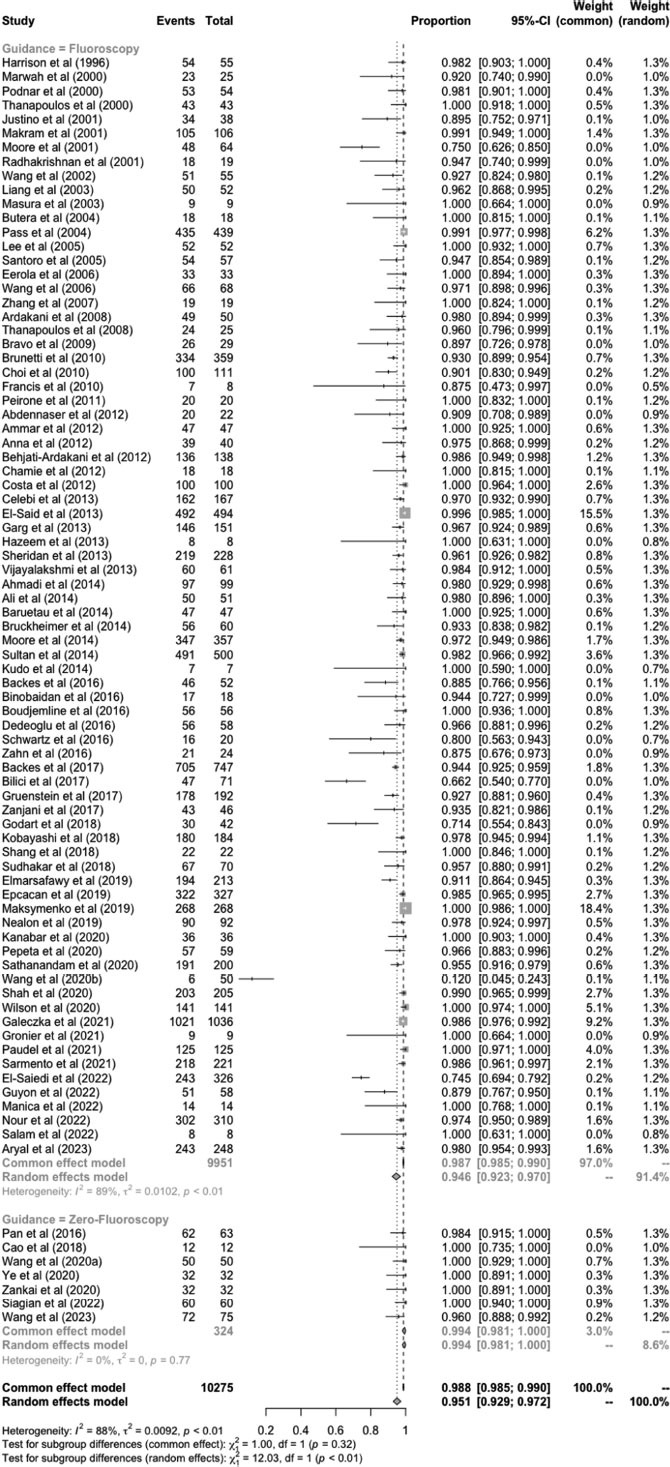
The success rate of transcatheter PDA closure.

## Data Availability

The data and supportive information are available within the article.

## LIST OF ABBREVIATIONS

PDA: Patent Ductus Arteriosus
PRISMA: Preferred Reporting Items for Systematic Review and Meta-Analysis
TEE: Transesophageal Echocardiography

## STANDARDS OF REPORTING

PRISMA guidelines and methodology were followed.

## SUPPLEMENTARY MATERIAL

PRISMA checklist and supplementary material are available on the publisher’s website along with the published article.
