# Supplementary material for: Safety and Efficacy of Zero Fluoroscopy Patent Ductus Arteriosus Closure in Comparison to the Standardized Fluoroscopy-Guided Procedure: A Systematic Review and Meta-Analysis
Source: Curr Cardiol Rev. 2025 Jan 31;21(5):E1573403X338573. doi: 10.2174/011573403X338573241101092849 (PMC12308002; doi:10.2174/011573403X338573241101092849)
Supplement: Supplementary file 1 — PRISMA checklist and supplementary material are available on the publisher’s website along with the published article. [file CCR-21-5-E1573403X338573_SD1.pdf]

Supplementary Table 1. Data Extraction of Included Studies

	Author (year)	Study design	Study location	Study interval	Total Patients	Patient gender (M/F)	Age [Median/Mean]	Weight (kg) [Median/Mean]	PDA characteristics		Device			Guiding Closure 2 Mei 2024	Follow-up	Underlying coexisting conditions (n)	Outcomes	
									Size (mm)	Type	Type	Size	Size details				Success / Fail	Complications (n)
1	**Harrison (1996)**	Cohort retrospective	Canada	August 1906 - December 1986	55	4 / 51	Mean: 38.3 ± 15.0	N/A	N/A	N/A	N/A	N/A	N/A	Fluoroscopy	1 year	N/A	54 / 1	N/A
2	**Marwah (2000)**	Prospective Cohort	India	August 1998 - May 1999	25	15/10	Median : 48 months (8 months - 26 years)	Median : 14 (4.8 - 48)	Ductal diameter :<br>Mean : 4.12 ± 1.51	N/A	The Amplatzer™ duct occluder, AGA Medical Corporation, Golden Valley, MN (25) [1]	N/A	N/A	Fluoroscopy	Range : 3 - 6 months	Muscular VSD (1), Pulmonary hypertension (15)	23 / 2	Intravascular hemolysis (1)<br>Loss of pedal pulse (1)
3	**Podnar (2000)**	Retrospective cohort	Slovakia	September 1996 and April 1999	54	N/A	Median coil: 0.7-28 (4.1)<br>Median ADO: 0.5-29 (4.9)	Median<br>Coil group: 7.7-69 (18)<br>ADO group: 6-58 (19)	Median<br>Coil:1.2-2.2 (1.7)<br>ADO: 1.9-7.5 (3.8)	Type A (19)<br>Type B (2)<br>Type E (5)	Detachable Cook PDA coils (26)<br>ADO (AGA Medical Corporation) [28]	N/A	N/A	Fluoroscopy	24 h, 1 month, 3 months, 1 year, and 2 years	pulmonary artery hypertension and failure to thrive (1).	Coil: 25/1<br>ADO: 28/0	0
4	**Thanopoulos (2000)**	Retrospective Cohort	Greece	May 1997 - February 1999	43	14/29	6.6 years (range 0.3 to 33 years) (SD: 6.55)	median weight was 20.3 kg (range 4.2 to 77 kg) (SD: 14.6)	Mean 3.9 ± 1.2 mm (range 2.2 to 8 mm)	type A (32), type B (4), type C (3), type D (2), type E (2)	ADO (43)	diameter : 6.1 ± 1.4 mm (range 4 to 10 mm)	N/A	Fluoroscopy	24 h, 1, 3, 6 months via color flow echocardiography	symptoms of heart failure and/or failure to thrive (10)	43/0	Trivial angiographic shunt through ADO (3)
5	**Justino (2001)**	Randomized trial	Canada	March 1995 - October 1997	Rashkind : 20<br>Gianturco : 18	Rashkind : 5/15<br>Gianturco : 9/9	Rashkind:<br>Median : 3.5 (2.2-17.6)<br><br>Gianturco:<br>Median : 4.7 (1.9-9.3)	Rashkind:<br>Median : 14.1 (10.5-47.5)<br><br>Gianturco:<br>Median : 18.9 (12.6-36.5)	PDA diameter :<br>Rashkind:<br>Mean : 1.9 ± 0.6<br><br>Gianturco:<br>Mean : 1.9 ± 0.6	Rashkind:<br>Type A (16)<br>Type D (1)<br>Type E (3)<br><br>Gianturco:<br>Type A (12)<br>Type D (1)<br>Type E (4)	Rashkind PDA Occluder, USCI Angiographics, C.R. BARD Inc., Billerica, Massachusetts (20)<br><br>Gianturco Coil (Cook, Inc., Bloomington, Indiana) (18) [2]	Mean:<br><br>Rashkind : 12 ± 0<br>Gianturco : 5.22 ± 1.11	Rashkind:<br>12 mm Rashkind (16)<br><br>Gianturco:<br>3 mm - 5 cm coil (1)<br>5 mm - 5 cm coil (13)<br>5 mm - 8 cm coil (2)<br>8 mm - 8 cm coil (2)	Fluoroscopy	Rashkind : 7.6 ± 3.3 months (Mean)<br>Gianturco : 10.6 ± 7.9 months (Mean)	N/A	Rashkind: 16 / 4<br>Gianturco: 18 / 0	Rashkind:<br>Persistent residual leaks (4)<br><br>Gianturco:<br>Embolization (2)<br>Loss of pedal pulse (1)<br>Residual leaks (4)
6	**Makram (2001)**	Retrospective Cohort	United States	N/A	106	29/77	7.1 ± 9.2 months (mean)	21 ± 18 kg (mean)	Median: 3.8 (1.2 - 8.1) mm	type A (85)<br>type B (4)<br>type C (6)<br>type D (3)<br>type E (8)	ADO-1 [106]	Mean Diameter: 7.96 ± 1.76<br>Mean Length: 7.2 ± 0.41	6/4: 32<br>8/6: 50<br>10/8: 15<br>12/10: 6<br>14/12: 1	Fluoroscopy	1 months	N/A	105/1	0
7	**Moore (2001)**	Clinical trial	USA	December 1996 - December 1999	62	62 [3]	Median : 36 (6-220) months	Median : 13.5 (4.1-79.1)	Min diameter :<br>Mean : 1.7 ± 0.74	N/A	Duct-Occlud device, PFM AG, Cologne, Germany (62) [4]	N/A	N/A	Fluoroscopy	Range : 2 - 12 months	N/A	48 / 14	Device embolization (3)
8	**Radhakrishnan (2001)**	Prospective cohort	India	N/A	19	10/9	Mean : 45 months (7-120 months)	Mean : 14.5 (7 - 23)	PDA diameter :<br>Mean : 5<br>Range : 4 -6.7	N/A	The Amplatzer™ duct occluder, AGA Medical Corporation, Golden Valley, MN (19) [5]	Mean: 9.22 ± 1.39	6/4 (1)<br>8/6 (6)<br>10/8 (10)<br>12/10 (1)	Fluoroscopy	Range : 3 - 6 months	Pulmonary hypertension (9)	18 / 1	Loss of femoral pulses (4)
9	**Wang (2002)**	Prospective cohort	Taiwan	Nov 1995 - Oct 2000	55	11/44	Median : 23 years (14-72 years)	N/A	Diameter of Ductus :<br>Mean :<br>≤ 3 mm : 2.2 ± 0.7<br>≥3.1 mm; ≤ 4 mm : 3.6 ± 0.3<br>> 4 mm : 5.3 ± 1.1 [6]	Type A (36)<br>Type B (8)<br>Type C (3)<br>Type D (2)<br>Type E (6)	Gianturco Coil, Cook, Inc., Bloomington, Indiana (55) [7]	N/A [8]	N/A	Fluoroscopy	Mean : 20.9 ± 10.2 months (18-42 months)	Down Syndrome (1)<br>Valvular aortic stenosis (1)<br>Hypertension (2)<br>Small VSD (1)<br>Mitral Regurgitation (1)<br>Pulmonary hypertension (9)	51 / 4	Device embolization (10)<br>Ventricular bigeminy (1)<br>Femoral artery hematoma (1)<br>Persistant residual shunt (4)
10	**Liang (2003)**	Prospective Cohort	Taiwan	March 1998 - May 2001	75	24/51	5.5 months (mean)	N/A	Mean Length: 7.2 ± 3.5 mm<br>Mean Diameter: 2.1 ± 1.0 mm	N/A	Gianturco Coil [75]	N/A	N/A	Fluoroscopy	every 3 months in 12 months	N/A	Group 1: 34 / 0<br>Group 2: 9 / 1<br>Group 3: 7 / 1	Residual shunt by single coil (1)<br>Residual shunt by multiple coils (1)
11	**Masura (2003)**	Cohort	Slovenia	January 2001 to April 2002,	9	9	Median: 5.2 (0.5 - 12.7 years)	Median: 20 (4.9 - 55 kg)	median: 2.5 (2.1-3.7 mm)	Type A (8)<br>Type C (1)	ADO (AGA Medical Corporation, Golden Valley, Minnesota) [9]	Mean Diameter: 7.77 ± 1,20 mm<br>Mean Length: 7,11 ± 0,33 mm	8/6 : 6<br>6/4: 2<br>10/8 : 1	Fluoroscopy	1, 6, and 12 months	N/A	9 / 0	0
12	**Butera (2004)**	Cohort	United States	March 2000 to March 2003	18	5/13	Mean: 1.51 ± 0.8 years	Mean: 9.2 ± 3.26	Mean: 3.68 ± 0.87	N/A	ADO (Aga Medical Corporation, Golden Valley, Minnesota, USA) (18)	Distal diameter Mean : 10.76 ± 1.30<br>Lenght Mean : 7 ± 0	8x6(5)<br>6x4(10)<br>10 x 8 (1)<br>5 x 4 (2)	Fluoroscopy	3,6,12, and 24 months	N/A	18/0	Linguna hematoma (2), Thrombosis (1)
13	**Pass (2004)**	Prospective cohort	USA	Sept 1999 - June 2002	439	141/298	Median : 1.8 years (0.2 - 70.7 years)	Median : 11 (4.5 - 164.5)	Min diameter :<br>Median : 2.6 (0.9 - 11.2)<br><br>Length :<br>Median : 7 (1.5 - 35)	Type A (328)<br>Type C (35)<br>Type E (52)<br>Not specified (24)	The Amplatzer™ duct occluder, AGA Medical Corporation, Golden Valley, MN (439) [9]	N/A	N/A	Fluoroscopy	Range : 6 - 12 months	Congestive heart failure (43)	435 / 4	Death (1)<br>Device embolization (2)<br>Partial obstruction of PA (2)<br>Pseudoaneurysm (2)<br>loss of femoral pulse (1)<br>Bleeding requiring transfusion (2)<br>Hypertension (1)<br>Hematoma of groin (7)<br>Arrhythmia (2)<br>Loss of peripheral pulse (6)<br>Other (7)
14	**Lee (2005)**	Cohort	Taiwan	July 1997 to June 2002	52	13/39	Mean: 2.89 ± 3.76 years	Mean: 11.92 ± 9.220	Mean: 2.34 ± 1.00 mm	Type A (32)<br>Type B (7)<br>Type C (6)<br>Type D (3)<br>Type E (4)	Gianturco coil (Cook Inc, Bloomington, IN, USA) (52)	N/A	N/A	Fluoroscopy	1,3,6,12, and 24 months	N/A	Single coil (41/0),<br>Multiple coil (11/0)	No complication
15	**Santoro (2005)**	Cohort retrospective	Italy	April 2000 - July 2004	57	N/A	Mean: 10.3 ± 16.0 years<br><br>Median: 3 years (4 mo - 67 years)	Mean: 30 ± 27<br><br>Median: 17 (4.5-94)	PDA diameter<br>Mean:<br>3.2 ± 1.2	type A (39)<br>type C (15)<br>type B (3)	Amplatzer Duct Occluder device (St. Jude Medical, Inc., St. Paul, MN, USA) (34)<br><br>multiple Cook detachable coils (20)<br>(Cook, Bloomington, IN, USA)	Mean: 6 ± 2 mm	N/A	Fluoroscopy	23 ± 12 months	pharmacologic therapy for CHF or recurrent PE (19)	54 / 3	failure to implant (2)<br>repeat embolization of multiple coils (1)
16	**Eerola (2006)**	Cohort	Finland	February 2003 and March 2004	33	13/20	median: 2.6 (0.9–10.6) years	median: 13.0 (6.9–32.8) kg	median: 1.5 (0.9 t-3.6) mm	N/A	Amplatzer PDA occlusion device (AGA, Golden Valley,<br>Minnesota [10])<br>Detachable coil ((Cook, Bloomington, Indiana [23])	N/A	N/A	Fluoroscopy	6 months	N/A	33/0	Minimal residual shunt (2)
17	**Wang (2006)**	Retrospective Cohort	Taiwan	April 2000 - June 2005	68	23/45	15.99 ± 21.71 months (mean)	28.32 ± 23.63 kg	Mean 4.1 ± 1.3 (range 2.5 to 8.5 mm)	type A (56)<br>type B (3)<br>type C (8)<br>type E (1)	ADO-1 [68]	Mean Diameter: 6.3 ± 1.6	N/A	Fluoroscopy	3 months	N/A	66/2	Distal embolization of the device (1)
18	**Zhang (2007)**	Retrospective Cohort	China	September 2000–May 2007	19	5/14	19.64 ± 21.68 months (mean)	29.25 ± 18.5 kg	Mean 3.1 ± 1.1 mm (range 2–6 mm)	type A (12)<br>type C (4)<br>type D (2)<br>type E (1)	ADO-1 [19]	Mean Diameter: 8.52 ± 1.47<br>Mean Length: 7.21 ± 0.41	6/4: 1<br>8/6: 14<br>10/8: 2<br>12/10: 2	Fluoroscopy	24 hours, 1 month, 2 months	frequent broncopulmonary infections (2); exertional dyspnoea (7); paroxysmal supraventricular tachycardia (2)	19/0	0
19	**Ardakani (2008)**	Cohort retrospective	Iran	May 2004 - March 2007	50	14/36	Mean: 6.11 ± 5.35	18.26 ± 14.06 (Mean)	Mean: 7.35 ± 2.57	N/A	Amplatzer<br>ductal occluder, AGA medical, Golder Valley, MN,<br>USA (50)	Mean: 7.56 ± 0.50	6/4 (8)<br>8/6 (14)<br>10/8 (17)<br>12/10 (7)<br>14/12 (2)<br>16/14 (1)<br>18/16 (1)	Fluoroscopy	Mean: 17.8 ± 9.88 months	failure to thrive (17)<br>mild aortic stenosis (2)<br>small VSD (1)<br>medium-sized VSD (1)<br>mild pulmonic stenosis (1)	49 / 1	distal embolization (1)<br>intraprosthetic residual shunt (5)<br>small immediate residual shunt (1)<br>significant blood loss (3)<br>thrombosis right femoral artery (7)<br>thrombosis left femoral artery (1)<br>unsuccessful heparin administered (4)<br>femoral thrombectomy (1)<br>mild inguinal hematoma (2)<br>left pulmonary branches obstruction (1)
20	**Thanopoulos (2008)**	Retrospective Cohort	Greece	January 2001 - May 2002	25	N/A	Not explicitly mentioned. 12 patients were 1-2 years old	range 7-12kg	Mean 4.8 ± 1.5 mm (range 3.8 to 8)	Not specified, had Type A, C, D, E	Amplatzer Duct Occluder (St. Jude Medical, Inc., St. Paul, MN, USA)	SSD: mean 6.5 ± 1.2<br>Plug Occluder: mean 6.8 ± 2.2 mm (range 4 to 11).	Swivel-disk device (SDD): 8/6 and 10/8 mm. Plug Occluder (PO): 7 or 8 mm	Fluoroscopy	Data for patients at 1 and 5 years after procedure	N/A	24/1	Hemolysis (1)
21	**Bravo (2009)**	Retrospective Cohort	Mexico	August 2005 and August 2008	29	8/21	mean 8.9 [2.8] months (4-12 months)	6.4 [1.5] kg (3.8-10kg)	Mean 3.16 ± 1.24 mm	type A (20), type B (1), type C (6), type E (2)	Amplatzer Duct Occluder (ADO), St. Jude Medical, Plymouth, Minnesota	mean distal diameter: 7mm (SD1.67), mean length: 6.90 (SD:0.72)	5/4 (3), 6/4 (14), 8/6 (9), 10/8 (2), 12/10 (1)	Fluoroscopy	1, 3, 6, 12 months using TTE	Pulmonary stenosis (1)<br><br>Interventricular communication (2)<br><br>Interatrial communication (2)<br><br>Aortic stenosis & vascular ring (1)<br><br>Down syndrome (5)<br><br>PH (17)	26/3	Device migration to bed of Descending Aorta (1), Femoral Artery Thrombosis (1), Increased Maximum Flow Velocity (1), Mild Stenosis in Left Pulmonary Branch (4)
22	**Brunetti(2010)**	Retrospective Cohort	United States	November 2005-September 2008	359	N/A	Mean: 4.3 ± 6.8 years	Mean: 18.7 ± 18.4 kg	N/A	Type A (206)<br>Type E (50)<br>Type C (53)<br>Type B (28)<br>Type D (22)	Gianturco (Cook Embolization Coils, Cook Cardiology, Bloomington, IN) [161]<br>ADO (AGA Medical,<br>Golden Valley, MN) [174]<br>Flipper Coil:18<br>Other: 6	Overall Mean<br>Diameter: 2.1 ± 1.4 mm<br>Length: 8.8 ± 3.6 mm	Gianturco (161)<br>Diameter: 1.3 ± 0.5<br>Length: 8.4 ± 3.4<br>ADO (174)<br>Diameter: 2.8 ±1.3<br>Length: 9.1 ± 3.8<br>Flipper Coil (18)<br>Diameter: 1.2 ± 0.6<br>length: 8.4 ± 2.6<br>Other (6)<br>Diamater: 6.5 ± 4.1<br>Length: 13.2 ± 2.5	Fluoroscopy	N/A	N/A	Gianturco coils: 156 / 5<br>ADO : 156 / 18<br>Flipper coils: 18 / 0<br>Other: 4 / 2	Embolization (9)<br>Transfusion (2)<br>Pulmonary hemorrhage (1)<br>PDA dissection (1)<br>Hematoma (1)<br>Prolonged bleeding without transfusion (1)<br>Peripheral pulse loss (1)<br>Arrhythmia (1)<br>Unable to implant device (2)
23	**Choi (2010)**	Cohort retrospective	South Korea	January 1996 - December 2007	111	31/80	Mean: 12.5±15.4	Mean: 27.0±20.0	Ductal diameter<br>Mean:<br>3.81±1.72	N/A	Sideris buttoned device (Custom Medical Devices, Athens, Greece; TX, USA) (7)<br><br>umbrella (USCI, Billerica, MA, USA) (5)<br><br>coils such as a Gianturco coil (Cook, Inc., Bloomington, IN, USA) (41)<br><br>Duct-occlud device (PFM, Cologne, Germany), and an ADO (AGA, MN, USA) (58)	N/A	N/A	Fluoroscopy	N/A	VSD (4)<br>ASD (2)<br>pulmonary valve stenosis (1)<br>anomalous origin of the LCA from pulmonary artery (1)<br>down syndrome (3)	100 / 11	left pulmonary artery narrowing (14)<br>arch obstruction (2)<br>transient bradycardia (1)<br>anemia requiring transfusion (2)<br>embolization device (1)<br>death (1)<br>significant hemolysis (1)

	Author (year)	Study design	Study location	Study interval	Total Patients	Patient gender (M/F)	Age [Median/Mean]	Weight (kg) [Median/Mean]	PDA characteristics	
									Size (mm)	Type
24	**Francis (2010)**	Retrospective Cohort	India	Jan 2002 - Aug 2008	8	N/A	Gestational Age: 28 ± 1.9 weeks (27-32 weeks) (SD: 1.88)	Median 1.1 kg (range 0.93 to 1.8 kg) (SD:280g)	Mean 2.5 ± 0.46 mm	N/A
25	**Peirone (2011)**	Cohort retrospective	Argentina	May - December 2010	20	5/15	Median: 4.7 years (6 months - 21 years)	Median: 16.4 (6-49)	mean diameter at pulmonary end<br>2.92 ± 0.61 mm<br><br>mean length<br>7.05 ± 1.17 mm<br><br>mean diameter of the aortic ampulla<br>9.52 ± 1.62 mm	type A (16), type E (2), type C (1), type D (1)
26	**Abdennaser (2012)**	Retrospective Cohort	Saudi Arabia	January 2000 - July 2010	22	5/17	5 ± 10.59 months (mean)	6.56 ± 1.25 kg (mean)	Median 4.5 (range 2-9) mm	type A (17)<br>type C (5)
27	**Ammar (2012)**	Prospective Cohort	Egypt	March 2009 and January 2011	47	16/31	Mean: 8.25 ± 4.16 months	Mean: 5.81 ± 2.32 kg	Mean: 3.8 ± 2.4 mm	N/A
28	**Anna (2012)**	Cohort	Brazil	March of 2011 to March of 2012	40	13/27	Median: 4.725 years (0.5-54.56)	Median: 17.3 (5-93)	Mean: 8.8 ± 3.4 mm	Type A (36)<br>Other (4)
29	**Behjati-Ardakani (2012)**	Prospective Cohort	Iran	May 2004 and October 2012	138	43/95	Mean: 3.53 ± 2.43 years	Mean: 11.9 ± 4.6 kg	Mean: 6.1 ± 1.87 mm	N/A
30	**Chamie (2012)**	Cohort	Brazil	March 2010 to December 2011	18	7/11	Mean: 13.7 ± 9.3 years	Mean: 2.9 ± 20.1	Mean: 4.2 ± 2.4 mm	Type A (10)<br>Type B (1)<br>Type E (7)<br>Other (1)
31	**Costa (2012)**	Observational	Brazil	2009-2011	80	32/48	Median 39.4 months (8.5 months to 170 months)	Median 14 kg (5–52 kg)	3.2 ± 1.1 mm	type A (78), type E (2)
32	**Celebi (2013)**	Retrospective Cohort	Turkey	March 2005 - May 2012	Overall: 167<br><br>ADO: 56<br>CDO: 111	48/119<br><br>ADO: 22/34<br>CDO: 26/85	Mean: 11.09 ± 13.88 years<br><br>ADO: 8.1 ± 11.9 years<br>CDO: 12.6 ± 14.6 years	Mean: 26.48 ± 23.91 kg<br><br>ADO: 31.6 ± 24.1 kg<br>CDO: 23.9 ± 23.5 kg	Mean: 5.94 ± 5.87 mm<br><br>ADO: 6.6 ± 1.6 mm<br>CDO: 5.6 ± 7.1 mm	N/A
33	**Chamie (2013)**	Retrospective Cohort	Brazil	March 2001- December 2002	33	9/24	Mean: 30.9 ± 12.8 years	Mean: 63.9 ± 12.4 kg	Mean 4.9 ± 2.8 mm	type A (25), type C (3), type D (2), type E (3)
34	**El-Said (2013)**	Cohort retrospective	Unites States	February 2007 - June 2010	496	177/319	Mean: 8.24 ± 7.68 years	Median: 12.2 (2.2 - 132.0)	lumen diameter<br>Median:<br>2.0 (0.5 - 12.0)	N/A
35	**Garg (2013)**	Retrospective Cohort	India	1st Aug 2004 - 31st May 2012	151	N/A	Median 2.7 years (2 months – 34 years)	Median 12.4 kg (2.2–58 kg)	1.8 mm to 14 mm (mean: 4.5 ± 2.3 mm)	N/A
36	**Hazeem (2013)**	Cohort retrospective	USA	January 2000 - April 2012	8	N/A	Median: 29.8 weeks (24-39)	Median: 1.43 (0.52-2.97)	Mean: 2.74 ± 0.47	Type A (1); Type C (5); Type E (2)
37	**Sheridan (2013)**	Retrospective Cohort	Australia	January 2003 - December 2011	228	N/A	median: 3.0 years (range; 0.4–16.4 years)	median 14.2 kg (range; 5.5–68 kg)	median 0.8 mm (range; 0.4–1.2 mm)	type A (153), type B (5), type C (20), type D (3), type E (22)
38	**Vijayalakshmi (2013)**	Retrospective Cohort	India	2003 - May 2012	61	25/36	8.9 months (mean)	5.3 kg (mean)	Range: 9 days - 12 months<br>Mean: 8.9 months<br>SD: 2.7	type A (46)<br>type B (6)<br>type C (4)<br>type D (1)<br>type E (4)
39	**Ahmadi (2014)**	Cohort retrospective	Iran	May 2007 - February 2012	99	34/65	Mean: 4 ± 3.2	N/A	PDA diameter of the ductus measured on the lateral or the 30-degree right anterioroblique view<br><br>Mean: 3.7 ± 0.9	N/A
40	**Ali (2014)**	Cohort retrospective	Egypt	March 2011 - September 2012	51	13/38	Median: 3 years (7 months - 35 years)	Median: 11 (5-80)	PDA size at pulmonary end<br>Mean: 3.6 ± 1.9<br><br>size of aortic ampula of PDA<br>Mean: 11.6 ± 4.9	type A (42)<br>type E (2)<br>type C (1)
41	**Baruteau (2014) [10]**	Prospective cohort	France	Dec 2009 - Dec 2014	47	38/9	Mean : 9.0 ± 6.5 months<br><br>Median : 7 (3-23) months [11]	Mean : 7.3 ± 2.9<br><br>Median : 6.9 (4.1-17) [12]	N/A [13]	Type E (34), Type C (13)
42	**Bruckheimer (2014)**	Retrospective cohort	Israel	June 2011 and December 2012	60	27/33	median: 3.2 years [0.6–15.8]	median: 14.5 kg [4–79]	Mean: 1.6 ± 0.4 mm	Type A (37)<br>Type C (19)<br>Type D (1)<br>Type E (3).

	Device			Guiding Closure 2 Mei 2024	Follow-up	Underlying coexisting conditions (n)	Outcomes	
	Type	Size	Size details				Success / Fail	Complications (n)
24	Gianturco Coil, Cook, Inc., Bloomington, Indiana	Mean wire thickness: 0.96 ± 0.03<br><br>Mean diameter: 4.63 ± 0.74	0.035-5-5 (1)<br>0.038-5-5 (3)<br>0.038-5-6 (1)<br>0.038-3-4 (4)	Fluoroscopy	9 months (range 3 to 72 months)	Tachypneic with signs of HF (8), ventilator (3)	7/1	LRTI death 6 months after coil closure (1)
25	Nit-Occlud® PDA-R (NOPDA-R; pfm Medical) (20)	Mean: 6.53 ± 1.05 mm	N/A	Fluoroscopy	N/A	N/A	20 / 0	0
26	Amplatzer Duct Occluder I [22]	Diameter: 7 ± 1.34<br>Length: 7.09 ± 0.29	6 x 4 : 13<br>8 x 6 : 7<br>10 x 8 : 2	Fluoroscopy	6 months, 12 months	N/A	20 / 2	N/A
27	ADOI (AGA Medical Corporation [35])<br>ADOII AGA Medical Corporation [12])	Mean Diameter: 6.48 ± 1.39 mm<br>Mean Length: 8.02 ± 1.79 mm	ADOI:<br>5/4 (1)<br>6/4 (18)<br>8/6 (14)<br>10/8 (2).<br>ADOII:<br>5/4 (9)<br>5/6 (3)	Fluoroscopy	1, 3, 6, and 12 months	Heart failure (44)<br>Failure to thrive (44)<br>Cardiomegaly (33)<br>Recurrent respiratory tract infections (21)	47/0	Minor complications:<br>Group A= 4<br>Group B = 2<br>Total Mild LPA Stenosis = 2<br>Intra-aortic bulge = 1
28	Amplatzer® Vascular Plug II (Abbott) (40)	Lenght: 9.4 ± 3.6 mm	N/A	Fluoroscopy	6 months	N/A	39/1	No complication
29	ADO (AGA Medical Corporation, Golden Valley, Minnesota [57])<br>ADO (Starway Medical Supplies Ltd., [81])	Mean Diameter: 7.4 ± 1.9 mm	6/4, 8/6, 10/8, 12/10, 14/12, 16/14, and 18/16.	Fluoroscopy	43.4 ± 23.5 months	N/A	136/2	Device embolization (2)<br>Thrombosis (4)<br>Mild inguinal hematoma (1)<br>Left pulmonary artery obstruction (1)<br>Left pulmonary artery stenosis (1)
30	PDA Occluder (CeraTM) (18)	Distal diameter Mean : 8.11 ± 2.61<br>Lenght Mean : 7 ± 0	CPO 6 (10)<br>CPO 8 (1)<br>CPO 10 (4)<br>CPO 12 (4)	Fluoroscopy	1 week	N/A	18/0	No complication
31	Amplatzer Duct Occluder I (St. Jude Medical, Inc., St. Paul, MN, USA)<br><br>Amplatzer Duct Occluder II (ADO II), St. Jude Medical, Plymouth, Minnesotta<br><br>Amplatzer Vascular Plug (AVP), St. Jude Medical, Golden Valley, Minnesota<br><br>Gianturco Coil, Cook, Inc., Bloomington, Indiana	N/A	N/A	Fluoroscopy	30 days, 6 months	N/A	100/0	Gianturco Coil Removal later reinserted(3), Nit-Occlud ® immediate highvelocity residual flow (2) observed, pneumothorax (1), Residual Flow (7)
32	Cardi-O-Fix duct occluder (CDO): 111<br>Amplatzer duct occluder (ADO): 56	Mean 9.87 ± 2.09<br><br>CDO: 9.8 ± 2.1<br>ADO: 9.9 ± 2.1	N/A	Fluoroscopy	6 months	N/A	ADO: 54/2<br>CDO: 108/3	pulmonary artery embolization (1)<br>pulmonary artery stenosis (4)<br>angle of the right ventricular outflow tract (5)<br>device protrusion to the aorta (2)
33	Flipper Coil (N/A), Amplatzer™ Duct Occluders type I (N/A), Amplatzer™ Duct Occluders type II (N/A), Cera™ PDA Occluders (N/A), Muscular VSD devices (N/A)	diameter: 8.3 ± 3.04,<br>length: 6.18 ± 1.40	Flipper coil: 6-4 (3), t-6 (3), 10-8 (5), and 12-10 (8)<br><br>ADO II prostheses: 5-4 (2) and 6-4 (1).<br><br>Cera™ PDA Occluders (CPO); 6-4 (2), 8-6 (2), and 12-10 (4)<br><br>12 mm Amplatzer™ Muscular VSD prosthesis Occluder (AMVSD) (1)<br><br>6-mm Cera™ Muscular VSD prosthesis Occluder (CMVSD) (1).	Fluoroscopy	46.1 ± 42.9 months (84.9% of patients)	dyspnoea on exertion (3)<br><br>severe PAH (functional class 3) (2)<br><br>mesocardia with left pulmonary sequestration (1)<br><br>deaf-mute (1)	**Success,** safe and effective with no major complications or deaths,all residual shunts closed within first month	Residual shunts immediately after procedure (2). In 6 , right femoral psudeoaneurysm (1)
34	Amplatzer Device (St. Jude Medical, Inc., St. Paul, MN, USA) (N/A)<br><br>coil group (N/A)<br>MReye (N/A)<br>Gianturco Coil, Cook, Inc., Bloomington, Indiana (N/A)<br>Flipper coils (FC), Cook Cardiology, IN, US (N/A)	N/A	N/A	Fluoroscopy	N/A	pulmonary hypertension (5)<br>isthmus narrowing (4)<br>unfavorable anatomy (2)<br>intracardiac disease (45)<br>genetic syndrome (65)<br>noncardiac problem (113)	492 / 4	pulmonary artery obstruction (1)<br>embolization or malposition (41)<br>temporary loss of pulse requiring heparin (6)<br>hematoma (6)<br>anesthesia related AE (5)<br>arrhythmias (3)<br>allergic reaction (1)<br>medication error (1)
35	Amplatzer Duct Occluders, Cardi-O-Fix Duct Occluders, Lifetech Duct Occluders	N/A	N/A	Fluoroscopy	1 month then regular intervals of 6 months until 3 years	N/A	146/5	Turbulence at LPA origin (8),Mild LPA origin stenosis (3), New-onset LPA turbulence (5), new-onset turbulence in descending aorta (2)
36	AVP II, Abbot, Santa Clara, CA (8)	Mean Diameter: 5.38 ± 1.6<br>Mean Length: 6.125 ± 0.83	6 mm AVP II (4)<br>4 mm AVP II (1)<br>3 mm AVP II (1)<br>10/8 ADO (1)<br>5/4 mm ADO (1)	Fluoroscopy	Median: 4.2<br>Mean: (1.3 ± 31.2) months	N/A	8 / 0	Cardiovascular compromise (1)
37	Flipper coils (FC) (Cook Cardiology, IN, USA) (76)<br><br>Amplatzer Duct Occluder I (ADO I), Amplatzer Duct Occluder II (ADO II), ADO II AS (St. Jude Medical, Inc., St. Paul, MN, USA) (132)	FC: N/A<br><br>ADO 1:<br>mean distal diameter: 7.72 mm (SD:0.92mm)<br>mean length: 7.05 (SD:0.21)	FC 5 mm–5 coil device was used in 59<br><br>ADO I<br>8 mm–6 mm device was used in 100 (75%), 6 mm–4 mm<br>device in 24 (18%) and 10 mm–8 mm device in 6 (4.5%)<br>patients	Fluoroscopy	8 months (range; 1–76 months) (FC), median three months (range; 1–75 months) (ADO)	N/A	219/9	Transient Vascular Complication (1)
38	ADO I and Lifetech duct occluder [47]<br>Modified duct occluder [5]<br>Angled duct occluder [3]<br>ADO II [5]<br>VSD muscular occluder [1]	Mean ADO-I<br>Diameter: 8.6 ± 1.43<br>Length: 7.36 ± 0.48<br>Modified duct occluder<br>Diameter: 10 ± 0<br>Length: 7 ± 0<br><br>Mean Angled duct occluder<br>Diameter: 12.67 ± 1.15<br>Length: 8 ± 0<br><br>Mean ADO-II<br>Diameter: 10.6 ± 1.14<br>Length: 5.45 ± 1.09<br>VSD muscular occluder:<br>Diameter:<br>Length:	ADO-I<br>12/10: 2<br>10/8: 15<br>8/6: 25<br>6/4: 5<br>Modified duct occluder<br>10/8: 5<br>Angled duct occluder<br>14/12: 1<br>12/10: 2<br>ADO-II<br>3/4: 1<br>5/4: 1<br>4/6: 1<br>5/6: 1<br>6/6: 1<br>VSD muscular occluder<br>7/7: 1	Fluoroscopy	N/A	Single kidney with hydronephrosis and hydroureter (1)	60/1	Device embolization (2)
39	Nit-Occlud PDA occlusion device (pfm, Germany) (59)<br><br>cook coil (6)<br><br>Amplatzer device (AGA) or Lifetech (34)	N/A	N/A	Fluoroscopy	N/A	N/A	97 / 2	embolization (1)
40	Amplatzer Duct Occluder (St. Jude Medical, Inc., St. Paul, MN, USA) (40)<br>Nit occlude PFM coil, N/A (5)<br>Amplatzer muscular VSD, (St. Jude Medical, Inc., St. Paul, MN, USA) (2)<br>Detachable Cook coil, N/A (4)	N/A	N/A	Fluoroscopy	13 ± 8.5 months	N/A	50 / 1	embolization (1)
41	Amplatzer Vascular Plug IV (AVP IV), St. Jude Medical, Golden Valley, Minnesota (47)	N/A	N/A	Fluoroscopy	Mean : 3.4 ± 1.4 years	Pulmonary Hypertension (12)<br>Down syndrome (1)	47 / 0	Device migration and embolization (1)
42	ADOIIAS (St Jude Medical, St. Paul, Minnesota [56])	Mean Diameter: 1.6 ±0.4 mm	N/A	Fluoroscopy	median: 2.3 months [0.1–17 months]	N/A	56/4	Small residual PDA (1)<br>Unstable position of device (3)<br>Device embolization (1)<br>Mild narrowing of the aortic isthmus (1)

	Author (year)	Study design	Study location	Study interval	Total Patients	Patient gender (M/F)	Age [Median/Mean]	Weight (kg) [Median/Mean]	PDA characteristics		Device			Guiding Closure 2 Mei 2024	Follow-up	Underlying coexisting conditions (n)	Outcomes	
									Size (mm)	Type	Type	Size	Size details				Success / Fail	Complications (n)
43	**Moore (2014)**	Prospective cohort	USA	Nov 2002 - October 2005	357	243/114	Median : 2.96 years (6 months - 21.9 years)	Median : 13.9 (4.7 - 109)<br>Mean : 18.1	Min Diameter :<br>Mean : 1.9<br>Median : 1.9 (0.5 - 3.9)	Type A (267)<br>Type B (17)<br>Type C (5)<br>Type D (18)<br>Type E (50)	Nit-Occlud PDA device, PFM Medical, Cologne, Germany (357) [14]	N/A	N/A	Fluoroscopy	Mean : 14.3 ± 3.78 months	Congenital heart defect (45)	347 / 10	Device related :<br>Device embolization (3)<br>Device protrusion to descending thoracic aorta (1)<br>False aneurysm of aorta (1)<br>Thrombus on pulmonary end of device (1)<br><br>Procedure related :<br>Temporary loss of pulse (2)<br>Excessive post-procedure hypoventilation (1)<br>Post-anasthesia nausea and vomiting (5)<br>Allergic skin reaction to tape (1)
44	**Sultan (2014)**	Cohort retrospective	Pakistan	January 2005 - December 2010	500	192/308	N/A	N/A	Mean: 4.5 ± 2.4	N/A	PDA Occluder device<br>Coils<br>ASD Occluder device	N/A	PDA Occluder:<br>5/4 (3)<br>6/4 (69)<br>8/6 (181)<br>10/8 (119)<br>12/10 (46)<br>14/12 (17)<br>16/14 (12)<br>18/16 (1)	Fluoroscopy	N/A	N/A	n= 491 / 9<br>PDA occluder: 448<br>Coils: 42<br>ASD occluder: 1	dislodged device (2)<br>faulty equipment (1)<br>pulse loss femoral artery for 6-24 hours (25)<br>pulse loss femoral artery >24 hours (10)<br>local hematoma (3)<br>ventricular tachycardia (2)
45	**Kudo (2015)**	Retrospective Cohort	Japan	N/A	7	3/4	28.8 ± 30.1 months (mean)	38.5 ± 14.7 kg (mean)	N/A	type A (7)	ADO-I [7]	Mean Diameter: 8.33 ± 2.94<br>Mean Length: 7.1 ± 0.4	6/4 : 2<br>8/6 : 3<br>14/12: 1	Fluoroscopy	N/A	71.2 years : atrial fibrillation, chronic renal dysfunction, was on anti-coagulan therapy	7/0	N/A
46	**Miguel (2015)**	prospective, multicenter, observational study	Argentina	June 2010 - February 2011	43	12/31	Median: 4.5 (1.4-18.4 years)	Median: 17.7 (10–67 kg).	Mean: 2.98 ± 1.03 mm	type A (36)<br>type B (5)<br>type C (1)<br>type E (1)	The Nit-OccludVRPDA-R trial [43]	Mean Diameter: 8.6 ± 2.8<br>Mean Length: 9.6 ± 3.4	N/A	Fluoroscopy	24 hrs, 1 month, 3 months	N/A	N/A	N/A
47	**Backes (2016)**	Retrospective Cohort	United States	January 2005 to January 2014	52	19/33	N/A	Median: 1.289 (0.475-1.887)	Median: 9.2 (4.4-20.6)	Type A (9)<br>Type C (39)<br>Type E (4)	Amplatzer Vascular Plug-II (St. Jude Medical, Saint Paul, Minnesota) (48), Amplatzer Ductal Occluder-I (St. Jude Medical, Saint Paul, Minnesota)(4)	AVP-II (Diameter Mean : 6 ± 1.49)<br><br>ADO(Distal diameter Mean : 9.75 ± 0.5, Lenght 7 ± 0)	ADO I:<br>5/4 (1)<br>6/4 (3)<br>AVP II:<br>4 mm (11)<br>6 mm (28)<br>8 mm (7)<br>10 mm (2)	Fluoroscopy	3 months	N/A	46/6	Aortic Obstruction (4), LPA bstruction (1), Embolized LPA (1)
48	**Binobaidan (2016)**	Cohort	Saudi Arabia	July 2013 - May 2015	18	7/11	median: 7 (4-23 months)	median: 8 (3.2–11 kg)	Mean<br>Diameter: 8.43 ± 1.61 mm<br>Length: 6.32 ± 0.97 mm	N/A	Amplatzer duct occluder additional size (ADOS, ST. Jude Medical corp. , St. Paul, Minnesota, USA) [10]<br>Amplatzer duct occluder type I (ADO I, ST. Jude Medical corp, St. Paul, Minnesota, USA) [8],<br>Occlutech (Occlutech, Jena, Germany) [18].<br>Amplatzer vascular plug type 2 (AVP2, ST. Jude Medical corp. , St.Paul, Minnesota) [18].	Mean Diameter: 5.11 ± 1.02 mm<br>Mean Length: 5.66 ± 1.53 mm	ADO I 6/4 (10)<br>ADOS 4/4 (8)	Fluoroscopy	N/A	Chronic renal impairment (2)<br>Relapse of leukemia (2)<br>Ventilated (1)	17 / 1	0
49	**Boudjemline (2016)**	Cohort	France	March 2014 to May 2015	56	21/35	Median: 1 years (0.8-24)	Median: 12.8 (3.1-60)	N/A	Type A (43)<br>Others (13)	PDA occluder (Occlutech®) (56)	Diameter : 10.1 ± 1.3<br>Lenght : 4.9 ± 0.7	5/3.5 (17)<br>6/4 (28) , 7/5 (4), 8/6 (6) and 10/8 (1).	Fluoroscopy	10 months (3-17.8)	N/A	56/0	No complication
50	**Dedeoglu (2016)**	Retrospective Cohort	Turkey	December 2013 - January 2016.	60	24/36	Median: 2.5 years (6 months - 35 years)	Median: 15 (5-60 kg)	Median 2.5 mm (1.2 - 11 mm)	type A (50)<br>type B (2)<br>type C (3)<br>type E (5)	the Occlutech PDA® occluder (ODO): 60	Mean Diameter: 11 ± 2.67<br>Mean Length: 5.72 ± 1.99	3/5: 1<br>3.5/5: 14<br>4/6: 17<br>5/7: 16<br>6/8: 7<br>8/10: 2<br>10/12: 2<br>14/18: 1	Fluoroscopy	7.6 months(range, 6–23 months)	N/A	56/2	Aorta embolization (1)
51	**Pan (2016)**	Prospective cohort	China	June 2013 - May 2015	63	27/37 [15]	Mean : 4.6 ± 2.9 years	Mean : 18.5 ± 7.5 kg	Min diameter :<br>Mean : 3.3 ±1.1	Type A (45)<br>Type C (13)<br>Type D (1)<br>Type E (4)	Amplatzer Duct Occluder II (ADO II), St. Jude Medical, Plymouth, Minnesotta (63) [16]	Mean: 4.6 ± 0.9	N/A	Zero Fluoroscopy	Mean : 13.5 ± 4.8 months	N/A [17]	62 / 1	0
52	**Schwartz (2016)**	Retrospective Cohort	United States	January 2020 - November 2014	20	8/12	median: 96 days (12– 247)	Median: 3.1 (1.7–4.7 kg)	Mean Diameter: 2.4 (1–5) mm<br>Mean Length: 10 (3–14) mm	type A (1)<br>type C (5)<br>type F (14)	Amplatzer Vascular Plug II [20]	N/A	N/A	Fluoroscopy	3 days, 7 days	airway abnormality: 5<br>evidence of left ventricular (LV) dilation by<br>LV end-diastolic volume: 10<br>had significant comorbid congenital heart disease consisting<br>of dextrocardia: 1	16 / 4	N/A
53	**Zahn (2016)**	Retrospective Cohort	United States	March 2013- February 2015	27	27	mean: 30 days (5-80 days) (SD:18.7 days)	1.2 kg (0.75 - 2.2 kg) (SD: 432g)	Median minimal diameter of 2.3 mm (range 1.3 to 3.5 mm)<br><br>Median length of 7.6 mm (range 5.8 to 10.6 mm)	type F tubular PDA	Amplatzer Vascular Plug (AVP), St. Jude Medical, Golden Valley, Minnesota	mean distal diameter: 3.90 mm (SD:0.995)<br><br>mean length 6mm (SD:0)	3 mm, n = 8; 4 mm, n = 10; 6 mm, n = 3	Fluoroscopy	12 h, 1 week, 1 month, 3 months, 6 months, 12 months, and then yearly	N/A	21/3	Device Malposition (2), LPA Stenosis (1)
54	**Backes (2017)**	Cohort	United States	January 2011 to March 2015	747	N/A	Median: 4.3 years(2.8 - 6.2)	Median: 4.6 (3.5 - 5.4)	N/A	Type A (276)<br>Type B (16)<br>Type C (316)<br>Type D (35)<br>Type E (88)<br>Others (16)	Amplatzer Ductal Occluder [ADO] I and II (St. jude) (N/A), Amplatzer Vascular Plug [AVP] (St.Jude Medical (N/A),Saint Paul (N/A), Minnesota(N/A))	N/A	N/A	Fluoroscopy	N/A	Left ventricular volume overload (486), Pulmonary hypertension (206), Bacterial endocarditis prevention (55)	705/42	Arrhythmia (13), Cardiac Reoperation(12), Major Bleeding (11)
55	**Bilici (2017)**	Retrospective Cohort	Turkey	September 2014 to August 2016	71	29/42	Median: 20.5 (6-194 months)	Median: 16 (6-68 kg)	Mean: 2.7 ± 0.9 mm	Type A (54)<br>Type B (1)<br>Type C (2)<br>Type E (14)	Occlutech duct occluder (Occlutech International AB, Helsingborg, Sweden) (71)	Mean Diameter: 4.5 ± 1.0 mm	N/A	Fluoroscopy	1, 3, 6 and 12 months	N/A	47/24	N/A
56	**Gruenstein (2017)**	Prospective cohort	USA	Aug 2008 - Apr 2011	192	72/120	Median : 2.5 Months	Median : 13.5	Max diameter:<br>Mean : 3.1 ± 1.2<br><br>Ductal length:<br>Mean : 7.6 ± 2.2	Type A (138)<br>Type B (2)<br>Type C (4)<br>Type D (10)<br>Type E (38)	Amplatzer Duct Occluder II (ADO II), St. Jude Medical, Plymouth, Minnesotta (192) [18]	Mean: 10.02 ± 1.05	4/3 (62)<br>6/3 (12)<br>4/4 (27)<br>6/4 (22)<br>4/5 (11)<br>6/5 (21)<br>4/6 (6)<br>6/6 (17)	Fluoroscopy	Range : 6 months - 2 years	Congestive heart failure (4)<br>Other congenital anomaly (46)	178/14	Procedural :<br>Device embolization (1)<br>Residual shunt (2)<br>Sinus tachycardia (1)
57	**Zanjani (2017)**	Retrospective cohort	Egypt and Iran	Nov 2011 - Feb 2016	46	18/28	Median : 1.5 years (6 months–13 years)	Median : 9.75 (5–32.7)	Max diameter :<br>Mean : 3.43 ± 2.5<br>Min diameter :<br>Mean : 0.98 ± 0.77	Type A (20)<br>Type C (13)<br>Type D (4)<br>Type E (6)	Nit-Occlud coils (NOCs), PFM AG, Cologne, Germany (46) [19]	Mean: 5.34 ± 1.01	4x4 (2)<br>5x4 (32)<br>6x5 (10)<br>7x6 (1)<br>11x6 (1)	Fluoroscopy	Median : 18 (1–50) months	Down Syndrome (2)<br>Glanzmann thrombobasthenia (1)<br>Milroy disease (1)<br>Syndactyly (1)<br>Pulmonary valve stenosis (3)	43 / 3	Coil embolization (2)<br>Detached coronary wire tip (1)<br>10mmHg pressure gradient in aorta (1)
58	**Cao (2018)**	Retrospective cohort	China	January and December 2015	12	N/A	Range: 12 to 35 years	Range: 30 to 65 kg	N/A	N/A	Amplatzer PDA device (Shan Dong Visee Medical Appa-<br>ratus Co. Ltd; China); and domestic PDA device [12]	Mean Diameter: 15.2±2.8 mm	N/A	Zero Fluoroscopy	1-2 years	Mild-moderate PH (11)	12 / 0	Transient arrhythmias (N/A)
59	**Godart (2018)**	Retrospective Cohort	France	April 2013 - September 2017	42	13/29	Median 34 months (range 4 months–68 years)	Median 12 kg (4.1–57 kg)	mean duct diameter was 3.76 mm<br><br>median 3.1 (1.69-9.95)	Krichenko type A (34), type E (6), type C (2)	Occlutech PDA Occluder, Occlutech, Helsingborg, Sweden	Mean disc diameter: 11.60 ± 2.62<br><br>Mean waist diameter: 5.24 ± 1.88<br><br>Mean shank diameter: 7.19 ± 1.93<br><br>Mean length: 6.15 ± 2.05 mm	4/6 device (n = 15)<br>5/7 device (n = 12)<br>6/8 device (n = 5)<br>3.5/5 device (n = 4)<br>10/12 device (n = 4)<br>8/10 device (n = 2)	Fluoroscopy	1, 6, 12 months (6-12 months follow up afterwards)	VSD (3)<br>ASD (1)<br>Aortic Regurgitation (1)<br>Scimitar Syndrome (1)<br>Trisomy 21 (7)<br>High BP (1)<br>Renal Failure (1)<br>Currarino Syndrome (1)	30/12	trivial-small residual shunt (12), right femoral artery thrombosis (3)
60	**Kobayashi (2018)**	Prospective Cohort	United States	2014-2017	184	126/58	Median: 3.4 [1.5 - 5.4 years]	Median: 14.1 [9.9-20.8 kg]	Mean Diameter: 8.0 ± 2.5 mm	Type A (154)<br>Type B (2)<br>Type C (4)<br>Type D (6)<br>Type E (18)	Nit-Occlud PDA device (PFM Medical, Cologne, Germany) [184]	Mean Diameter: 7.11 ± 1,86<br>Mean Length: 4.32 ± 0,78	4/4 :11<br>5/4 : 23<br>6/5 : 39<br>7/6 : 53<br>9/6 : 39<br>11/6 : 15	Fluoroscopy	2 months, 12 months, 24 months	History of congenital heart disease other than PDA (44)	180 / 4	Procedure-related (15)<br>Device-related (6)<br>Not device or procedure-related (102)
61	**Shang (2018)**	Randomized controlled trial	China	July 2014 - September 2015	22	12/10	Mean: 15.8 ± 15.1 years	Mean: 34.7 ± 20.1	Mean: 5.3 ± 1.6	N/A	MemoPart™ PDA Occluder, Lepu Medical Tech (12)<br>SHSMA PDA Occluder, Lepu Medical Tech (10)	Overall Mean: 10.46 ± 2.61<br><br>Diameter<br>MemoPart™ PDA Occluder: 10.17 ± 2.33<br>SHSMA PDA Occluder: 10.8 ± 3.01	MemoPart™ PDA Occluderl: 8 mm (5); 10 mm (3); 12 mm (2); 14 mm (2)<br><br>SHSMA PDA Occluder: 6 mm (1); 8 mm (1); 10 mm (5); 14 mm (2); 16 mm (1)	Fluoroscopy	6 months	N/A	22 / 0	Instant residual leaks (16); Transient arrhythmia (16); Persistent residual leaks (1)
62	**Sudhakar (2018)**	Retrospective Cohort	India	January 2011 - February 2017	70	20/50	Mean: 23 ± 11 years	-	Mean 4.8 ± 2.2	type A1 (44), type A2 (7), type B1 (2), type B2 (1), type C (6), type D (2), type E (4)	Cook's detachable coils (4), occluders (ADO-I and II, Lifetech, Cardi-O-Fix) (64), vascular plug (1) and ventricular septal occluder device (1).	N/A	N/A	Fluoroscopy	1-6 months, 1-5 years	Systemic Hypertension (5), Diabetes (4), Associated cardiac lesions (15), LV systolic dysfunction (4)	67/3	N/A

	Author (year)	Study design	Study location	Study interval	Total Patients	Patient gender (M/F)	Age [Median/Mean]	Weight (kg) [Median/Mean]	PDA characteristics		Device			Guiding Closure 2 Mei 2024	Follow-up	Underlying coexisting conditions (n)	Outcomes	
									Size (mm)	Type	Type	Size	Size details				Success / Fail	Complications (n)
63	**Elmarsafawy (2019)**	Retrospective Cohort	Egypt	January 2008 - December 2016	213	116/97	mean: 9.2 ± 5.2 months	mean: 7.6 ± 1.8 kg	Mean 2.81 ± 0.51 mm	type A (198), type B (13), type C (2)	Nit-Occlud Coil Occluder (pfm Medical, Am Söterberg, Nonnweiler-Otzenhausen, Germany) (107)<br><br>Amplatzer Duct Occluder I (St. Jude Medical, Inc., St. Paul, MN, USA) (84)<br><br>Nit-Occlud® coil PDA-R (NOPDA-R; pfm Medical) (22)	N/A	N/A	Fluoroscopy	1, 3, 6, 12 months via Echocardiography	Isolated lesion (132)<br>ASD (55)<br>VSD (14)<br>Pulmonary valve stenosis (10)<br>Aortic valve stenosis (2)	194/19	Projection of Occluding Device into Aorta (3), Mild LPA Obstruction (2), Significant Blood Loss (4)
64	**Epçaçan (2019)**	Cohort retrospective	Turkey	May 2010 - July 2018	327	123/204	Mean: 7.33 ± 7.67	21.29 ± 15.62 (Mean)	Mean: 3.74 ± 2.14	type A (224)<br>type B (11)<br>type C (25)<br>type D (9)<br>type E (56)	Amplatzer duct occluder I (St. Jude Medical, Inc., St. Paul, MN, USA)<br>Amplatzer vascular plug II (St. Jude Medical, Inc., St. Paul, MN, USA)<br>Amplatzer muscular ventricular septal occluder (St. Jude<br>Medical, Inc., St. Paul, MN, USA)	N/A	N/A	Fluoroscopy	Mean: 28.29 ± 17.15 months	Down syndrome (1)<br>Pulmonary hypertension (189)	322 / 5	transient loss femoral artery pulse (10)<br>device embolization (5)<br>slight protusion of AVP II device without significant stenosis (1)<br>immediate protusion device required surgical device removal (1)
65	**Maksymenko (2019)**	Cohort retrospective	Ukraine	July 2008 - December 2015	268	N/A	Mean: 6.6 ± 5.5 years<br><br>Median: 5.2 years (0.4-62)	Mean: 24.1 ± 14.3<br><br>Median: 19.3 (5.5-97)	diameter<br>median: 1.5 mm (0.4-4)<br><br>ampulla<br>median: 5 (1-15)<br><br>length<br>median: 9 (2-25)	Type E (118), Type A (89), Type B (9), Type C (21), Type D (24), type F (7)	Nit-Occlud® coil PDA-R (NOPDA-R; pfm Medical) (268)	minimal diameter<br>1.5 (0.5-4) (median)<br><br>ampulla<br>5 (1-15) (median)<br><br>length<br>9 (2-25) (median)	N/A	Fluoroscopy	N/A	additional cardiac anomalies (40)	268 / 0	thromboembolytic event (1)
66	**Nealon (2019)**	Cohort	United States	January 2003 and February 2017	92	42/50	Median: 3.0 years (0.5-11.1)	Median: 1.196 (0.475 - 4.165)	N/A	Type A (19)<br>Type B (40)<br>Type D (18)<br>Type E (15)	Flipper coil (N/A), Amplatzer Vascular Plug-I (N/A),Amplatzer Vascular Plug-II (6), Amplatzer Ductal Occluder (3)	AVP-II (Diameter Mean : 6 ± 1.49)<br>ADO(Distal diameter Mean: 9.75 ± 0.5, Lenght 7 ± 0)	N/A	Fluoroscopy	28 Weeks	N/A	90/2	LPA Obstruction (22), DAObstruction (10), Residual shunting (2)
67	**Kanabar (2020)**	Cohort retrospective	India	2003 - 2018	36	17/19	Mean: 16.6 ± 12.5	Mean: 39.62 ± 16.7	Median: 14 (12-20) mm	type A (23)<br>type B (2)<br>type C (11)	Cocoon duct occluder (20), Cera duct occluder (13), Cera muscular ventricular septal defect occluder (MVSDO) (2) (Lifetech Scientific, Shenzhen, China)<br><br>Amplatzer atrial septal occluder (ASO, AGA Medical Corporation, Golden Valley, Minnesota, USA) (1)	Mean waist diameter<br>25.78 ± 5.59<br><br>Mean waist length<br>8.67 ± 1.87	18/16 (13)<br>20/18 (14)<br>24/22 (1)<br>24 ASO (1)<br>24 (Cera MVSDO) (2)<br>30/28 (5)	Fluoroscopy	N/A	N/A	36 / 0	partial obstruction of PA (1)<br>aortic obstruction (1)<br>hemolysis due to residual shunt (1)
68	**Pepeta (2020)**	Cohort prospective	South Africa	June 2011 - June 2017	59	27/32	Median: 4 months (23 days - 12 months)	Median: 3.6 (0.9 – 5.8)	PDA narrowest diameter<br>median: 1.9 mm (1.0–3.4 mm)<br><br>Ductal ampulla<br>median: 6.1 (2.8–10.6)<br><br>PDA length<br>median: 8.8 (3.7–18.4)<br><br>Descending aortic diameter<br>median: 5.3 (3.6–10.6)	type A (16)<br>type C (15)<br>type D (4)<br>type E (24)<br>type B (0)	Amplatzer Duct Occluder typetwo additional sizes (ADO II AS) (Abbott Laboratories, St Jude Medical, St Marks, Minnesota) (92)	Mean: 6.1 (2.8–10.6)	N/A	Fluoroscopy	2 years (15 months - 2 years)	secundum ASD (1)	57 / 2	device embolization (3)<br>blood transfusion (4)<br>bleeding at puncture site (2)<br>catheter induced SVT (1)
69	**Sathanandam (2020)**	Prospective cohort	USA	June 2017 - Feb 2019	200	102/98	Mean : 3.92 ± 33.74 months	Mean : 6.25 ± 10.77	Max diameter:<br>Mean : 4.6 ± 1.5<br><br>Ductal length:<br>Mean : 10.4 ± 2.9	Type A (49)<br>Type B (3)<br>Type C (28)<br>Type D (5)<br>Type E (18)<br>Type F (91)<br>Others (6)	Amplatzer Piccolo Occluder, Abbott Structural Heart, Plymouth, MN (200) [20]	Mean: 5.58 ± 0.76	3/2 (12)<br>3/4 (5)<br>4/2 (76)<br>4/4 (30)<br>4/6 (1)<br>5/2 (28)<br>5/4 (29)<br>5/6 (10)	Fluoroscopy	6 months	None	191 / 9	Procedural :<br>Device embolization (5)<br><br>Post-Procedural :<br>Aortic obstruction (2)<br>Device migration (2)<br>tricuspid valve regurgitation (5)<br>Blood loss (2)<br>Hemolysis (1)
70	**Shah (2020)**	Cohort retrospective	India	2008 - 2015	205	74/131	Mean: 7.92 ± 3.61	Mean: 16.68 ± 10.82	ductal diameter on aortic angiogram<br>Mean:<br>5.6 ± 2.43 mm	N/A	ADO-I (AGA Medical, Golden Valley, Minnesota, USA) occluder (205)	N/A	N/A	Fluoroscopy	94.36 ± 21.23 months	N/A	203 / 2	device-induced aortic obstruction (1)<br>left pulmonary stenosis (1)<br>groin hematoma (3)<br>transient loss of peripheral pulse (27)<br>trainsient LV dysfunction that required medication (5)<br>residual shunting (9)<br>transient hemolysis (6)
71	**Wang (2020)**	Prospective cohort	China	May 2015 - May 2017	TTE : 50<br>Fluoroscopy : 50	TTE : 12/38<br>Fluoroscopy : 15/35	TTE: 10.5 ± 12.7 years (Mean)<br>fluoroscopy: 15.2 ± 16.4 years (Mean)	TTE: 29.6 ± 19.9<br>fluoroscopy: 32.5 ± 23.1	Max diameter :<br>TTE:<br>Mean : 4.8 ± 1.3<br><br>fluoroscopy:<br>Mean :4.7 ± 2.2	N/A	PDA occluder, Shape Memory Alloy Co., Ltd, Shanghai (100) [21]	Mean:<br><br>TTE: 10.5 ± 1.4<br>fluoroscopy: 10.2 ± 2.3	N/A	Zero Fluoroscopy	Median : 12.0 months (range, 10.0–15.5 months)	None	TTE : 49/1<br>Fluoro : 50/0	TTE :<br>Residual shunt (resolves in 24 hour) (4)<br><br>Fluoroscopy :<br>Residual shunt (resolves in 24 hour) (4)<br>Femoral artery hematoma (2)
72	**Wilson (2020)**	Retrospective Cohort	Canada	June 2001 - June 2017	141	33/108	43±15 years (mean)	han	Mean Diameter: 4.1 ± 1.9 mm<br>Mean Length: 10.0 ± 4.7 mm	type A (104)<br>type B (3)<br>type C (7)<br>type D (2)<br>type E (25)	ADOI (136)<br>Amplatzer Muscular VSD: 3<br>Amplatzer Septal Occluder: 1<br>Amplatzer Vascular Plug 4: 1	Mean Diameter: 9,35 ± 1,77<br>Mean Length: 7,56 ± 0,65	ADOI<br>5 x 4: 2<br>6 x 4: 7<br>8 x 6: 50<br>10 x 8: 52<br>12 x 10: 25<br>Amplatzer Muscular VSD<br>6 mm :1<br>10 mm: 1<br>18 mm: 1<br>Amplatzer Septal Occluder (ASO)<br>13 mm: 1<br>Amplatzer Vascular Plug 4<br>6 mm: 1	Fluoroscopy	7 months	Symptomatic (dyspnea on exertion, chest<br>pain, palpitations, or dizziness): 64<br>Heart failure: 4<br>Endocarditis. 1	141 / 0	Groin hematoma (1)
73	**Ye (2020)**	Retrospective Cohort	China	March 2018 to February 2020	32	8/24	Mean: 5.12 ± 1.32 years	Mean: 17.32 ± 3.6	Mean Type A (8.5 ± 2.7)<br>Mean Type B (4.2 ± 1.2)	Type A (20)<br>Type B (12)	ADOII (AGA Medical Corporation, Golden Valley, MN) (32)	N/A	N/A	Zero Fluoroscopy	1 and 3 months	N/A	32/0	No complication
74	**Zankai (2020)**	Prospective Cohort	Beijing	March 2018 to February 2020	32	8/24	Mean: 5.12 ± 1.32 years	Mean: 17.32 ± 3.6 kg	N/A	Type A (20)<br>Type C (12)	ADOII (AGA Medical Corporation) [32]	Mean Diameter: 4.8 ± 2.3 mm.	N/A	Zero Fluoroscopy	1 month, 3 months, 6 months	Peripheral facial paralysis (1)<br>Epilepsy (1)<br>Multiple cervical deformities (1)	32/0	0
75	**Gałeczka (2021)**	Cohort retrospective	Poland	October 1993 - February 2020	1036	364/672	Median: 4 years (2 months - 84.5 years)	Median: 17 (3.9 - 136)	PDA diameter<br>Median: 2 mm (1-10 mm)<br><br>PDA length<br>Median: 7 mm (2-20 mm)	type A (508)<br>type B (25)<br>type C (55)<br>type D (156)<br>type E (273)	Rashkind Device PDA Occluder, USCI Angiographics, C.R. BARD Inc., Billerica, Massachusetts (25)<br>detachable coil, N/A (469)<br>DO I Amplatzer (St. Jude Medical, Inc., St. Paul, MN, USA) (159)<br><br>DO I Chinese copies, (Lifetech Scientific, Shenzhen, China)<br>: Cardi-O-Fix (63), Hyperion (37), HeartT (32), Cera duct occluders (9)<br><br>ADO II, N/A (32)<br>ADO II AS, N/A(209)<br>ASO Amplatzer, N/A (2)<br>ASO CardioSEAL/STARFlex devices, N/A (5)<br>(St. Jude Medical, Inc., St. Paul, MN, USA)<br><br>Amplatzer muscular VSD occluder, Abbot, Santa Clara, CA (6)<br>Amplatzer Vascular Plug type II (St. Jude Medical, Inc., St. Paul, MN, USA) (4)	N/A	N/A	Fluoroscopy	N/A	N/A	1021 / 15	failed implantation due to :<br><br>unstable position of device (3)<br>embolization (7)<br>protrusion risk (1)<br>duct morphology (1)<br><br>left pulmonary artery wall dissection (1)<br>spontaneous closed duct (2)
76	**Gronier (2021)**	Retrospective Cohort	France	Nov 2019 - Mar 2021	9	3/6	Mean 5.3 ± 3.4	Mean 4.1 ± 1.0 (2.9–5.8) kg (SD: 0.84)	mean minimal ductal diameter of 5.6 ± 1.0 (4.5–7.0) mm<br><br>mean ductal length of 4.3 ± 0.6 (3.5–5) mm,	Krichenko type C (4), Krichenko type B (5)	Konar MF VSD Occluder, Life Tech	Mean disc diameter: 11.56 ± 1.67<br><br>Mean waist length: 4 ± 0	6/4 (4)<br>7/5 (2)<br>8/6 (1)<br>9/7 (1)<br>10/8 (1)	Fluoroscopy	Median 12 (6–25) months	N/A	9/0	N/A

	Author (year)	Study design	Study location	Study interval	Total Patients	Patient gender (M/F)	Age [Median/Mean]	Weight (kg) [Median/Mean]	PDA characteristics		Device			Guiding Closure 2 Mei 2024	Follow-up	Underlying coexisting conditions (n)	Outcomes	
									Size (mm)	Type	Type	Size	Size details				Success / Fail	Complications (n)
77	**Paudel (2021)**	Cohort retrospective	United States	January 2015 - December 2019	125	66/59	Median 29 days (8-48)	Median 900 (600 - 1460 gram)	Mean diameter at PA end<br>TTE: 3.1 ± 0.72<br>fluoroscopy: 3.2 ± 0.94<br><br>Mean diameter at aortic end<br>TTE: 4.5 ± 0.68<br>fluoroscopy: 4.4 ± 0.85<br><br>Mean length EL2 (alternate technique)<br>TTE: 11.0 ± 1.83 mm<br>fluoroscopy: 10.8 ± 2.15 mm<br><br>Mean length EL1 (conventional technique)<br>TTE: 8.2 ± 1.73<br>fluoroscopy: 10.8 ± 2.16	N/A	N/A	N/A	N/A	Zero Fluoroscopy and Fluoroscopy	N/A	N/A	125 / 0	N/A
78	**Sarmento (2021)**	Retrospective Cohort	Portugal	Jan 2006 - Sep 2018	221	N/A	mean age of 5.6 years (4 months-19 years)	-	N/A	N/A	Nit-Occlud PDA device, PFM Medical, Cologne, Germany (139)<br><br>Amplatzer Duct Occluder (ADO) (St. Jude Medical, Inc., St. Paul, MN, USA) (79)<br><br>Amplatzer Vascular Plug (AVP), St. Jude Medical, Golden Valley, Minnesota (3)	Mean Nit-Occlud coil:<br>distal diameter: 5.73 ± 1.56<br>mean length 3.83 (SD:0.58)<br><br>Mean ADO 1:<br>distal diameter 6.93 (SD: 1.37)<br>mean length 6.77 (SD:0.70)<br><br>Mean ADO 2:<br>distal diameter 4.56 (SD: 1.50)<br>mean length 5.7 (SD0.67)	Nit-Occlud® coil: 4x4 (31), 5x4 (45), 6x5 (27), 7x6 (22), 9x6 (12), 11x6 (2)<br><br>ADO I: 5x4 (9), 6x4 (26), 8x6 (31), 10x8 (1), 12x10 (1)<br><br>ADO II: 3x4 (1), 3x6 (3), 5x6 (1), 6x6 (4)	Fluoroscopy	1, 3, 6, 12 months, then anually (Global average follow-up was 49 months)	Previous duct closure (2)	218/3	Device Embolization after 48 hours (1), Loss of Arterial Pulse (3)
79	**El-Saiedi (2022)**	Retrospective Cohort	Egypt	N/A	323	224/99	Median 24 months (7-156 months)	Median 11kg (5-103kg)	Mean 0.84 ± 0.47 cm	Krichenko type A (245), Type E (43), Type C (25), type D (10), type B (3)	Amplatzer Duct Occluder I (ADO I), St. Jude Medical, Inc., St. Paul, MN, USA (136)<br><br>Amplatzer Duct Occluder II (ADO II), St. Jude Medical, Plymouth, Minnesotta (6)<br><br>Amplatzer Vascular Plug II (AVP II), St. Jude Medical, Golden Valley, Minnesota (6)<br><br>Flipper coils (FC), Cook Cardiology, IN, USA (62)<br><br>Amplatzer Muscular VSD Occluder (mVSDO), Lifetech Scientific, Shenzhen, China, Amplatzer Septal Occluder (ASO), Lifetech Scientific, Shenzhen, China (5)<br><br>Nit-Occlud PDA device, PFM Medical, Cologne, Germany(73)	N/A	ADO I (136)<br><br>ADO II (32)<br><br>AVP II (6)<br><br>mVSDO & ASO (5)<br><br>PFM (73)<br><br>Flipper (62)	Fluoroscopy	1, 3, 6 months	N/A	243/83	Immediate Residual Shunt (83), Residual Shunt 10 Min after (47), Residual Shunt 1 Month after (15), Residual Shunt 6 months after (7), Device embolization (3), Loss of femoral pulsation (13), protrusion into LPA (8), Protrusion to desc ao (7), Hemolysis (12)
80	**Guyon (2022)**	Cohort retrospective	United States	June 2018 - May 2021	58<br><br>MVP: 25<br>Microplug: 25<br>Piccolo: 8	MVP: 11/14<br>Microplug: 10/15<br>Piccolo: 4/4	Mean: 0.93 ± 0 months<br><br>MVP: 0.87 ± 0<br>Micro Plug: 1 ± 0<br>Piccolo: 0.93 ± 0	Mean: 1.44 ± 0 kg<br><br>MVP: 1.51 ± 0<br>Micro Plug: 1.40 ± 0<br>Piccolo: 1.31 ± 0	Mean PDA aortic end: 3.59 ± 0<br><br>MVP: 3.9 ± 0<br>Micro Plug: 3.4 ± 0<br>Piccolo: 3.2 ± 0<br><br>Mean PDA size minimum: 2.37 ± 0<br><br>MVP: 2.5 ± 0<br>Micro Plug: 2.3 ± 0<br>Piccolo: 2.2 ± 0	N/A	MVP, Medtronic, Minneapolis, MN (25)<br>Microplug, KA Medical, Minneapolis, MN (25)<br>Amplatzer Piccolo Occluder, Abbot, Santa Clara, CA (8)	N/A	N/A	Fluoroscopy	5 months	N/A	MVP (21 / 4)<br>MicroPlug (25 / 0)<br>Piccolo (5 / 3)	MVP: Embolization (2), Aortic obstruction (1), suboptimal positioning (1)<br>Piccolo: Embolization (1), suboptimal positioning (2)
81	**Manica (2022)**	Cohort prospective	Brazil	March 2020 - February 2021	14	N/A	Mean: 38.85 ± 17.35 days	Mean: 1.41 ± 0.41	diameter mean:<br>3.0 ± 0.67<br><br>length mean:<br>6.9 ± 2.12	N/A	Amplatzer ADO IIAS (Abbot Structural Heart, Plymouth, MN) (11)<br><br>Piccolo (Abbot Structural Heart, Plymouth, MN) (3)	N/A	N/A	Fluoroscopy	N/A	mechanical ventilation pre-procedure (11)<br>bronchopulmonary dysplasia (6)<br>acute kidney injury (1)<br>anuria (1)<br>duodenal atresia (1<br>tracheosophageal fistula (1)<br>pulmonary valve stenosis (1)<br>Edwards syndrome (1)<br>pulmonary artery hypertension (1)	14 / 0<br><br>but 3 deaths are not related to the procedure	need pulmonary valvuloplasty (1)<br>drop in systemic saturation due to tricuspid insufficiency (2)
82	**Nour (2022)**	Cohort retrospective	Egypt	June 2017 - January 2021	308	113 / 195	Median: 2.7 years (3 months - 20 years)	Median: 11.8<br>(5 - 90)	Mean: 2.86 ± 0.96	Type A (250); Type B (8); Type C (48)	ADO I, AGA Medical Corporation, Golden Valley, MN (132);<br>Hyperion (25);<br>Occlutech PDA Occluder, Occlutech, Helsingborg, Sweden (24);<br>Lifetech PDA Occluder, Life Tech(12);<br>Amplatzer muscular VSD, Abbot, Santa Clara, CA (10);<br>ADO II, AGA Medical Corporation, Golden Valley, MN(4)	Median: 6 mm (6 - 12 mm)	N/A	Fluoroscopy	1 months, 3 months, 6 months, 1 year	N/A	302 / 8	Embolization (4); Hemolysis (2); Minor vascular complication (3); Benign arrhythmia (3); Device encroachment on ascending aorta or LPA (3)
83	**Salam (2022)**	Cohort retrospective	United Kingdom, France	2017 - 2019	8	N/A	Median: 6.3 months (1-18 months)	Median: 5.3 (2.4 - 8.2)	PDA width<br>Mean: 4.43 ± 1.13<br><br>PDA length<br>Mean: 8.17 ± 3.54	type C (8)	Occlutech® delivery set (Occlutech International AB, Helsingborg, Sweden) (3)<br><br>180° TorqVue™ Amplatzer® delivery system (Abbott, St. Paul, MN, USA) (5)	Mean: 36.50 ± 16.06 mm	N/A	Fluoroscopy	28 months (14-41 months)	pulmonary hypertension (7)<br>Mowat Wilson syndrome (1)<br>T21 (2)<br>Cat eye syndrome (1)	8 / 0	mild left pulmonary artery stenosis (2)
84	**Siagian (2022)**	Prospective cohort	Indonesia	March 2019 - April 2020	60<br><br>Fluoroscopy: 30<br>Echocardiography: 30	Fluoroscopy: 6/24<br>Echocardiography: 14/16	Median<br>Fluoroscopy: 2.8 (1.8-20.3)<br>Echocardiography: 6.0 (2.4-9.0)	Median<br>Fluoroscopy: 12.9 (8-40.3)<br>Echocardiography: 17 (12.2-23)	Fluoroscopy Mean (5.58 ± 1.97)<br>Echocardiography Mean (5.07 ± 2.25)	Type A	**Fluoroscopy:** Memopart PDA Occluder, Lepu Medical Tech (16); Konar MF VSD Occluder, Life Tech (8); HeartR PDA Occluder, Life Tech (3); ADO I, AGA Medical Corporation, Golden Valley, MN (2); ADO II, AGA Medical Corporation, Golden Valley, MN (1)<br><br>**Echocardiography:** Memopart PDA Occluder, Lepu Medical Tech (1); PDA Occluder, Life Tech (4); Konar MF VSD Occluder, Life Tech (8); ADO II, AGA Medical Corporation, Golden Valley, MN (10): Multifunctional Occluder, Life Tech (7)	Diameter Mean:<br>Fluoroscopy: 16.5 ± 3.7<br>Echocardiography: 13.4 ± 3.5	N/A	Zero Fluoroscopy	48 hours	N/A	60 / 0	Instant residual leaks (13); Persistent residual leaks (1)
85	**Aryal (2023)**	Cohort retrospective	Nepal	October 2017 - March 2020	311	76/172	Mean: 50.2 ± 41.9 months	Mean: 13.9 ± 8.3	N/A	Type A (311)	N/A	N/A	N/A	Fluoroscopy	N/A	N/A	retrograde transarterial: 147 / 5<br>Venous only: 96 / 0	N/A
86	**Wang (2023)**	Cohort retrospective	China	January 2019 - September 2021	75	33/42	Mean: 2.95 ± 1.75 years	Mean: 10.25 ± 6.75	Mean: 6.5 ± 2.6	Type A (12); Type C (63)	SHSMA PDA Occluder, Lepu Medical Tech (75)	N/A	N/A	Zero Fluoroscopy	12 months	N/A	72 / 3	0
